# Artificial intelligence-informed mobile mental health apps for young people: a mixed-methods approach on users’ and stakeholders’ perspectives

**DOI:** 10.1186/s13034-022-00522-6

**Published:** 2022-11-17

**Authors:** Christian Götzl, Selina Hiller, Christian Rauschenberg, Anita Schick, Janik Fechtelpeter, Unai Fischer Abaigar, Georgia Koppe, Daniel Durstewitz, Ulrich Reininghaus, Silvia Krumm

**Affiliations:** 1 Department of Psychiatry II, University of Ulm and BKH Guenzburg, Lindenallee 2, Guenzburg, 89312 Ulm, Germany; 2 Department of Forensic Psychiatry and Psychotherapy, University of Ulm and BKH Guenzburg, Ulm, Germany; 3 Department of Public Mental Health, Central Institute of Mental Health, Medical Faculty Mannheim, Heidelberg University, Mannheim, Germany; 4 Department of Theoretical Neuroscience, Central Institute of Mental Health, Medical Faculty Mannheim, Heidelberg University, Mannheim, Germany; 5 Department of Psychiatry and Psychotherapy, Central Institute of Mental Health, Medical Faculty Mannheim, Heidelberg University, Mannheim, Germany; 6 Centre for Epidemiology and Public Health, Health Service and Population Research Department, Institute of Psychiatry, Psychology & Neuroscience, King’s College London, London, UK; 7 ESRC Centre for Society and Mental Health, King’s College London, London, UK

**Keywords:** Youth mental health, Mental health promotion and prevention, Mobile health (mhealth), Public mental health, Ecological momentary assessment, Ecological momentary interventions, Artificial intelligence

## Abstract

**Background:**

Novel approaches in mobile mental health (mHealth) apps that make use of Artificial Intelligence (AI), Ecological Momentary Assessments, and Ecological Momentary Interventions have the potential to support young people in the achievement of mental health and wellbeing goals. However, little is known on the perspectives of young people and mental health experts on this rapidly advancing technology. This study aims to investigate the subjective needs, attitudes, and preferences of key stakeholders towards an AI–informed mHealth app, including young people and experts on mHealth promotion and prevention in youth.

**Methods:**

We used a convergent parallel mixed–method study design. Two semi–structured online focus groups (n = 8) and expert interviews (n = 5) to explore users and stakeholders perspectives were conducted. Furthermore a representative online survey was completed by young people (n = 666) to investigate attitudes, current use and preferences towards apps for mental health promotion and prevention.

**Results:**

Survey results show that more than two-thirds of young people have experience with mHealth apps, and 60% make regular use of 1–2 apps. A minority (17%) reported to feel negative about the application of AI in general, and 19% were negative about the embedding of AI in mHealth apps. This is in line with qualitative findings, where young people displayed rather positive attitudes towards AI and its integration into mHealth apps. Participants reported pragmatic attitudes towards data sharing and safety practices, implying openness to share data if it adds value for users and if the data request is not too intimate, however demanded transparency of data usage and control over personalization. Experts perceived AI-informed mHealth apps as a complementary solution to on–site delivered interventions in future health promotion among young people. Experts emphasized opportunities in regard with low-threshold access through the use of smartphones, and the chance to reach young people in risk situations.

**Conclusions:**

The findings of this exploratory study highlight the importance of further participatory development of training components prior to implementation of a digital mHealth training in routine practice of mental health promotion and prevention. Our results may help to guide developments based on stakeholders’ first recommendations for an AI-informed mHealth app.

**Supplementary Information:**

The online version contains supplementary material available at 10.1186/s13034-022-00522-6.

## Background

Mental illness is the leading cause of disease burden in young people aged 10–24 years [1]. Studies show that three–quarters of mental disorders emerge in adolescence, by the age of 24 [2]. Hence, mental health promotion and mental disorder prevention in young people represent important societal responsibilities [3]. Whilst mental health providers offer various therapeutic and advisory services, it is well documented that young individuals have the poorest access to mental health services among all age groups [4]. Research therefore calls for an increase in public mental health interventions to help prevent mental health problems and build resilience in young people [5, 6]. Due to the ubiquitous nature of smartphones and young peoples’ frequent use of technologies, mobile mental health (mHealth) apps have emerged as a promising way for mental health promotion and prevention in youth [7–9]. Especially in times of increased burden like the COVID–19 pandemic [10–12], studies show that there is an objective need for apps that address prevention and early intervention in youth [13]. Mobile apps come with the advantages of accessibility, ease of use and anonymity [14], but the development of apps appropriate to the needs and preferences of young people is still facing several challenges [15]. Existing apps available in major app stores rarely use evidence–based frameworks [16–19], frequently do not involve young people in the development [20], and therefore often remain insufficiently tailored to the needs of young users [4, 20, 21].

Advances in Artificial Intelligence (AI) could pave the way for better personalization and novel approaches of digitization in mental health care [22, 23]. Algorithms may support personalization by collecting multimodal mobile data using Ecological Momentary Assessment (EMA) and, on this basis, determine predictive (risk) factors, temporal dynamics of mental health trajectories and translating them back into suggestions for behavioral options. EMA encompasses diverse ambulatory assessment techniques (eg, diaries, physiological information) to capture real-time data concerning internal and external cues (eg, situational variables) [24, 25]. EMIs on the other hand are defined by Heron & Smyth as “treatments that are provided to people during their everyday lives (ie, in real time) and in natural settings (ie, real world)” [25], thus including a variety of ambulatory treatment strategies [24, 25].

EMA and EMIs are therefore ‘ecological’ because they are provided in natural settings and are ‘momentary’ because they are delivered to users in real-time [25]. Especially the combination of EMIs and EMA with deep learning algorithms may enable mHealth apps to provide effective mental health promotion and mental disorder prevention to users. While EMIs deliver customized training components adjusted by EMA to the person's current state of health and experienced stress in real time [26, 27], deep learning based algorithms promise improved outcome predictions and more individually tailored selection of intervention components. Employed in mHealth apps for prevention and intervention, Recurrent Neural Networks (RNNs) have shown to forecast depressed mood based on self-reported histories [28]. RNNs are able to adapt to individual behavior through continuous parameter updating, and to optimize the allocation of individual training components via dynamic feedback and learning loops [29, 30]. Apps that use RNNs with EMIs and EMA may therefore allow for more adaptive, real–time, and real–world deployment of interventions in individuals’ daily lives, thus offering a low–threshold mental health promotion and mental disorder prevention tailored to person, moment, and context.

Randomized controlled trials that investigate the efficacy of interventions in this novel approach for prevention remain necessary [31–33]. Rauschenberg et al. found in an uncontrolled pilot EMI study, that within 3 weeks of using a compassion focused program (including, inter alia, EMA–informed interactive tasks), stress sensitivity, momentary negative affect, and psychotic experiences could be reduced in ten individuals aged 14–25 [34]. Although these findings need to be replicated in a well–powered RCT, the study shows promising results. The study also indicated feasibility and safety, including high satisfaction rates, low burden of app usage and no adverse effects in participants [34]. In addition to clinical validation, more research is required in order to explore user perspectives in context of AI-informed (or RNN–based) mHealth apps using EMIs and EMA. In a systematic review, investigating attitudes and perspectives of patients towards clinical artificial intelligence–defined as “any software made to automate intelligent behavior in a health–care setting for the purpose of diagnosis or treatment” [35]–authors concluded that even though patients and the general public conveyed openness towards AI, many reservations as well as a preference for human supervision remained. The authors also highlighted knowledge deficits and participants’ perception of risks and weaknesses of AI (eg, risks to privacy, risks due to self–treatment, data fraud and profit seeking) [35]. Although selection criteria only encompassed tools for AI-informed diagnosis or treatment of patients, close proximity in attitudes towards AI-informed EMI/EMA apps can be assumed, as they become as deeply entangled in young people’s everyday lives. However, in context of mHealth apps, research on users’ perceptions has primarily focused on apps that don’t rely on machine learning algorithms or the use of multimodal data (eg, in form of EMA, sensor data) [25, 36, 37]. MHealth apps with a more sophisticated technical approach, such as the personalized delivery of intervention components based on AI-involvement, may therefore elicit different concerns and preferences in users.

Along with ensuring that mHealth apps are tailored to specific user needs through novel technical approaches, young people should also be involved in the development of mental health apps in order to ensure that their needs are met. Following the International Declaration on Youth Mental Health [38], the participation of young people in the entire development processes of mental health services (eg, mHealth apps) is deemed crucial to make sure that such services are aligned to their understanding of mental health and wellbeing. While participatory research improves the validity and relevance of findings [39], only a few studies fully consider the perspective of young people when developing apps [23, 40, 41]. In addition to the perspectives of young people, experiences and perspectives of other key stakeholders (eg, school psychologists, pedagogues) should also be taken into account, when novel technical approaches in their field of expertise are investigated [42, 43]. The aim of this exploratory study was therefore to inform future participatory app development through first insights in subjective perspectives of young individuals and key stakeholders regarding their concerns, needs, and preferences for an AI-informed mHealth app for mental health promotion and mental disorder prevention and to complete results with a representative sample of young people.

## Methods

### Research objectives

With the goal of increasing participation of youths in the developing processes of mHealth apps, this mixed-methods study aims to investigate the subjective perspectives of young people and key stakeholders, towards their concerns, needs, and preferences in an AI-informed mHealth app and to guide the future participatory development of the app. To this end, the following questions were addressed:

1. What are the subjective views on mental health and mHealth apps by experts and young people?
2. How do young people currently make use of mHealth apps?
3. What knowledge and opinions do young people express about AI?
4. What opportunities and risks do young people and experts perceive towards the use of AI for personalized intervention components?
5. What are recommendations of young people and experts for an AI–informed mHealth app at an early stage of development?

### Study design

In order to provide insights into subjective views and to examine the utilization of mHealth apps, we used a convergent parallel mixed–method study design [QUAL + QUAN] [44–46]. Qualitative data was collected by means of focus groups with young people and expert interviews with mental health experts (ie, school psychologists, pedagogues, media specialists, mHealth app developers). A representative online survey was conducted for quantitative data acquisition. In line with convergent design procedure, respective analysis was conducted independently for each data collection method. Results were subsequently integrated by comparing and illustrating them in order to identify points of convergence or divergence. Through the consolidation of results, we aimed to explore the generalizability of our findings. Both study phases were conducted in German and results were later translated to English.

### Study setting

This study formed part of the living lab “AI4U–Artificial Intelligence for personalized digital mental health promotion and prevention in youth” [13, 47]. Living labs aim for cooperation between science and society and therefore involve actors from business, civil society, and science in order to test and research innovative models of action [48]. The living lab AI4U (study period: 01/2021–12/2023) aims to develop, evaluate, and implement of a mobile, AI–informed intervention in routine public mental health promotion and mental disorder prevention in young people [49]. The planned app’s overall purpose is to strengthen emotional resilience and other protective factors for mental health in youth. Following a participative, transdisciplinary research approach, the study involves the target population (young people between 12 and 25 years), key stakeholders including school psychologists, psychological counsellors, media experts, and representatives of the digital industry as well as an interdisciplinary research group (AS, CG, CR, DD, GK, JF, SK, UFA, UR). The participatory approach calls for the early involvement of relevant stakeholders in the development of an AI–informed mHealth app. Results presented are part of the preparatory stage of AI4U (March 2020 to December 2020), in which we aimed to analyze the target group, the role of key stakeholders, and framework conditions for the structural embedding of an AI–informed mHealth app. The Consolidated Criteria for Reporting Qualitative Research Checklist [50] was used to ensure explicit and comprehensive reporting on how qualitative research was conducted (see Additional file 5).

### Focus groups and expert interviews

#### Recruitment

Participants for interviews with key informants were recruited from the steering committee of the AI4U living lab. Steering committee members were invited via email. For the recruitment of focus groups, we again reached out to steering committee members for recommendations, and as a result contacted a mentoring training program for high school students (“Diversity Coach Program”) and one school affiliated with a steering committee member (Youth Foundation BW). Due to active lockdown measures few students in the respected organizations responded to recruitment measure, whereas students for the focus groups with young people were therefore recruited via personal networks using a snowball system. Participants received invitation emails including study information and contact details. Prior to interviews and focus groups, the participants were provided with written study information, an informed consent form (approved by ethics committee), and a participant information sheet about study procedures (research reasons and goals, assumptions, and information about the interviewers/facilitators). Only participants who provided written informed consent were included. In addition, for under–aged focus group participants, we obtained informed consent from parents or legal guardians.

#### Data collection

Data collection started in March 2020. Focus groups and expert interviews were conducted online due to active lockdown measures in place because of the COVID–19 pandemic, using a video–based online tool from the Central Institute for Mental Health (ZI) in Mannheim, Germany. Both focus groups and expert interviews were audio recorded and transcribed verbatim. Solely the participants and respective facilitators/interviewers attended these meetings. The interviewers had no previous relationship with participants before the study commenced. Field notes were taken before and after data collection by CG and CR.

Focus groups were held on March 25th and April 16th 2020 and lasted approximately 1.5 h. Each focus group was led by scientific team members with experience in facilitating focus groups (facilitator: CG; co–facilitator: CR). Semi–structured topic guides were used (see Additional file 1). After a short introduction round (1), participants were asked about their understanding of mental health and what they already do to promote theirs (2). We asked participants if they had already used mHealth apps for this purpose. Depending on their level of knowledge, CR explained the project’s definition of mHealth apps:

*“Health apps are apps that can help you deal better with everyday stress and worries, support you to be more physically active or allow you to work on or monitor your habits/behaviors (eg, your sleep quality, exercise)."*

Subsequently, the use of AI in everyday life and the young peoples’ knowledge about it was explored (3) followed by a short presentation about the concept of mHealth apps and RNNs in the planned app. The co-facilitator also described some of the basic information about EMIs and EMA to participants of the focus groups. After providing feedback on the planned app and possible concerns (4), young people were asked to provide recommendations and suggest desirable functionalities of the app, and whether they were interested in further participation (5).

Expert interviews were conducted online between March 18th and April 30th 2020 by CG & CR. All expert interviews lasted between 1 and 1.5 h. We used semi–structured topic guides (see Additional file 2). Themes addressed (1) mental health promotion in the experts’ professional context, (2) relevance of apps in mental health, (3) opportunities, limits, and concerns regarding the planned mHealth app, and (4) opportunities for further participation for practitioners. In advance, experts were briefed during a steering committee meeting about the planned mHealth app. Here, all of its relevant functions were explained and the use of EMIs and EMA briefly discussed.

#### Data analysis

Following a qualitative approach, data was analyzed by CG following structural content analysis according to Kuckartz [51, 52]. In a first step, important parts of the transcripts were highlighted, and texts were read repeatedly to get familiar with the data. Main thematic categories were developed in line with the topic guides. Memos and codes were generated for important aspects prior to further assessment, allowing for the discovery of the previously unknown themes and data. After coding all the material with main categories, relevant passages of the transcripts were analyzed and inductive subcategories were developed. Categories and sub–categories were consecutively added to the evolving coding tree and themes were reviewed, refined, modified, and structured. We checked categories for disjunctions and summarized them when necessary. Results and an interim report were discussed in the research team (CG, CR, SK, UR). All qualitative data analyses were performed using MAXQDA version 12.

### Survey

#### Recruitment

Using a cross–sectional panel study, a representative sample of 16–25 year–old young people was recruited from the German general population. Participants were internet users interested in participating in surveys or polls and thus registered members of the Norstatpanel by Norstat Deutschland GmbH. Norstatpanel is certified according to ISO 26362 and ISO 9001 standards and operates in accordance with relevant data protection laws (eg, General Data Protection Regulation (GDPR)). We obtained informed consent from the sample by Norstat. Selection of participants was conducted at random and invitations for participation were sent via email. Incentives for participation were either paid directly (ie, around 0.10€ per minute) or through other benefits (eg, discounts for shopping). We used quotas for gender, educational background, as well as population density according to public available data of the Federal Statistical Office of Germany, to ensure sample representativeness. We opened the online survey on May 7th, and concluded data collection on May 16th, 2020. Some of the survey's findings regarding the COVID-19 pandemic were previously published [13].

#### Measures

In total, we covered 30 questions in 6 major thematic blocks, excluding items used to assess socio–demographics. The survey assessed (1) technology use and respective ownership, (2) use of technologies for health promotion and monitoring, (3) utilization of mHealth apps, and (4) attitudes towards the use of AI for personalized intervention components, (5) COVID-19–related cognitive preoccupation, worries, and anxiety, and (6) current use of, and attitudes toward, mHealth apps. Some of the items were based on a survey investigating willingness to share data in outpatient mental health services [53], as well as studies assessing the acceptance of mHealth apps across several populations [42, 54]. Most of the items, however, were constructed by the multidisciplinary research team to ensure alignment with the survey's specific objectives and to best inform the development of a machine —learning–based mHealth app for mental health promotion and prevention. To ensure standardized assessment, we provided participants with the following definition of an mHealth app:

*“Health apps are apps that can help you deal better with everyday stress and worries, support you to be more physically active or allow you to work on or monitor your habits/behaviors (eg, your sleep quality, exercise)."*

Similarly, for questions concerning the application of AI to personalize intervention components, we illustrated its functionality utilizing an everyday example:

*“Every day–often without noticing it – we use so–called Artificial Intelligence, or AI in short. When we search for information on YouTube, Spotify Music or Google, AI always plays an important role. Just like us humans, AI systems are able to evaluate large amounts of information in order to make decisions. And not only that: just like us, AI systems can learn over time. For this, the AI needs as much information/data as possible.”*

Depending on the respective question, answer options included 6–point Likert scales, but also multi–answer or open–ended questions (see Additional file 3). A more detailed description of measures as well as COVID-19–related topics can be found elsewhere [13].

#### Statistical analysis

We used descriptive statistics to report on the major sample characteristics as well as relevant variables. All analyses were performed with STATA version 15.1.

## Results

### Overview

Following a convergent parallel mixed–method study design we analyzed quantitative and qualitative data separately and chose a joint display in the results section for a comprehensive overview over our major findings. Since qualitative data allows for new themes to emerge and existing themes to be confirmed or discarded through the participants, we used the 4 major themes identified in qualitative research and complemented findings with quantitative data (see Table 1 for an overview).

**Table 1:** Overview over mixing of results

Themes	Subthemes	Mixing of results
Concepts of mental health	Young peoples’ understanding of mental health	Qualitative only
	Experts’ understanding of mental health in youth	Qualitative only
Current use of mHealth apps in young people	–	[QUAL + QUAN]
AI use in the context of mHealth apps	Young people on AI in everyday life	[QUAL + QUAN]
	Opportunities and risks seen by young people	[QUAL + QUAN]
	Opportunities and risks seen by experts	Qualitative only
Recommendations for AI–informed mHealth apps with EMA/EMIs	Young peoples’ recommendations in focus groups	[QUAL + QUAN]
	Experts’ recommendations	Qualitative only

### Sample characteristics

#### Expert interviews and focus groups

Two focus groups included a total of 8 participants aged 15 to 20 years (M_age_ = 18.6 years) with 4 females and 4 males (dropouts: N = 0). A total of 5 expert interviews (2 psychologists, 2 pedagogues, and 1 representatives of the digital industry) were conducted. Five out of 6 steering committee members agreed to participate at this time (see Additional file 4). With one expert, we could not arrange an interview appointment within a reasonable timeframe.

#### Survey sample

In our representative online survey, 666 individuals, aged between 16 and 25 years (M_age_ 21.3) with a representative gender distribution were included (Table 2). A more detailed description of socio-demographics of the sample can be found elsewhere [13]. From 685 youths that completed the online survey, 19 individuals were excluded after completion of quality control checks (eg, implausible response time or pattern).

**Table 2:** Demographic data including age, gender, educational level and ethnic background of participants (N = 666)

Characteristic	Participants
**Age (years), mean (SD; range)**	21.3 (2.6, 16–25)
**Gender, n (%)**	
Female	318 (47.8)
Male	346 (52.0)
Divers	2 (0.3)
**Educational level, n (%)**^a^	
Low	135 (20.3)
Middle	358 (53.7)
High	173 (26.0)
**Migrant/ethnic minority group position, n (%)**	
1st generation migrant	53 (7.9)
2nd generation migrant	156 (23.4)

^a^Educational levels were defined as follows: ‘low’ (ie, lower secondary school certificate, secondary school certificate, no school-leaving qualification, or visiting respective school types), ‘middle’ (ie, high-school diploma, completed vocational training, or visiting respective school type/doing an apprenticeship), ‘high’ (ie, bachelor’s, master’s degree, or currently studying)

### Concepts of mental health

#### Young peoples’ understanding of mental health

From young people’s perspectives, mental health mostly meant successful coping with challenges (8/8). Thus, being “mentally healthy”” implied being able to cope with challenges or crises in a successful manner:

"*It* [mental wellbeing; CG] *doesn't necessarily mean that you're always in a good mood and always just happy, but that if things aren't going so well, you don't crash completely, because somehow you’re just strong enough to get out of there again.*" [P3, FG1]

Young people in focus groups also highlighted conflicts with themselves, parents or friends, as well as work—or school—related issues as the most challenging circumstances for their mental health (4/8). One female participant underlined technology and media consumption to be a source of stress for her mental health, especially the constant distraction by messages in social media [P1, FG2]. For half of the young people, “mental health” also meant awareness of one’s physical and mental wellbeing, including a better understanding of the functioning of one’s mind. One male participant stated: “*I think, very often you are depressed or sad when you just can't figure out what makes you sad.“* [P4, FG1].

Focus group participants described a wide range of activities to promote and sustain their mental health. Most common were sporting activities (8/8), alone time (4/8), meditation or meditative activities such as drawing or writing (3/8), and social contacts, for example with friends or family (4/8). Participants also underlined paying attention to sleep and waking times or keeping track of their nutrition. With regard to distancing oneself from technology, one female participant emphasized to regularly "refrain from using cell phones and screens and such things" (P1, FG2). At this point, none of the participants had explicitly addressed the use of mHealth apps to promote their mental health.

#### Experts’ understanding of mental health in youth

In general, experts had a broad understanding of mental health in young people (eg, confidence, self–regulation of feelings or resilience). One expert emphasized the importance of behaviors that complement mental health, such as enough sleep, eating regularly, avoiding too much screen time, social contacts, exercise, and/or leisure activities (EXP1).

Concerning mental health in the planned app, two experts (EXP4, EXP2) raised concerns about using the terms “mental health”, “mental health promotion” or “prevention”, given their semantic proximity to “illness”. In order to avoid reinforcing the binarity of being healthy vs. ill, they recommended using the term “fitness”:

*“You have a kind of continuum from what extends from health to non–health. And not necessarily a hard transition. And that's why I think it might be helpful not to put this label of health or illness on such an application, but rather* [to focus; CG] *on fitness.”*[EXP4]

All experts underlined the high future relevance of digital interventions for mental health promotion and prevention due to a low threshold access to for young people, affinity of youths to digital media, and the inevitability of future developments in technology. Therefore, non–commercial providers should engage in app development as well as to:

*“…pick up on this trend, the power that is in there and use it productively and maybe leave out a few things that you would do if you were to use it as a business idea, which should then be highly profitable.”*[EXP3]

However, two mHealth experts reported few to no mentions of apps in communication with colleagues or young people (2/2). Yet the current COVID–19 pandemic was seen as an opportunity for change [EXP4, EXP5].

### Current use of mHealth apps in young people

When asked for the use of mHealth apps in our focus groups (with a broad definition of mHealth apps, also including mental health promoting physical activity), the majority of young people (7/8) reported first–hand experiences. Participants reported the usage of sports and fitness apps (eg, “Adidas Sport App”, “Freeletics”; 3/8), meditation and mindfulness apps ("Calm", "Waking Up," “Headspace", etc.; 3/8), apps for sleep optimization ("Sleep Cycle" and others; 2/8), and nutrition apps (“My–Fitness–Call”; 1/8). This is in line with the survey results where more than two–thirds of young people reported to have experience with such apps (only 30% never used one).

**Fig. 1:**
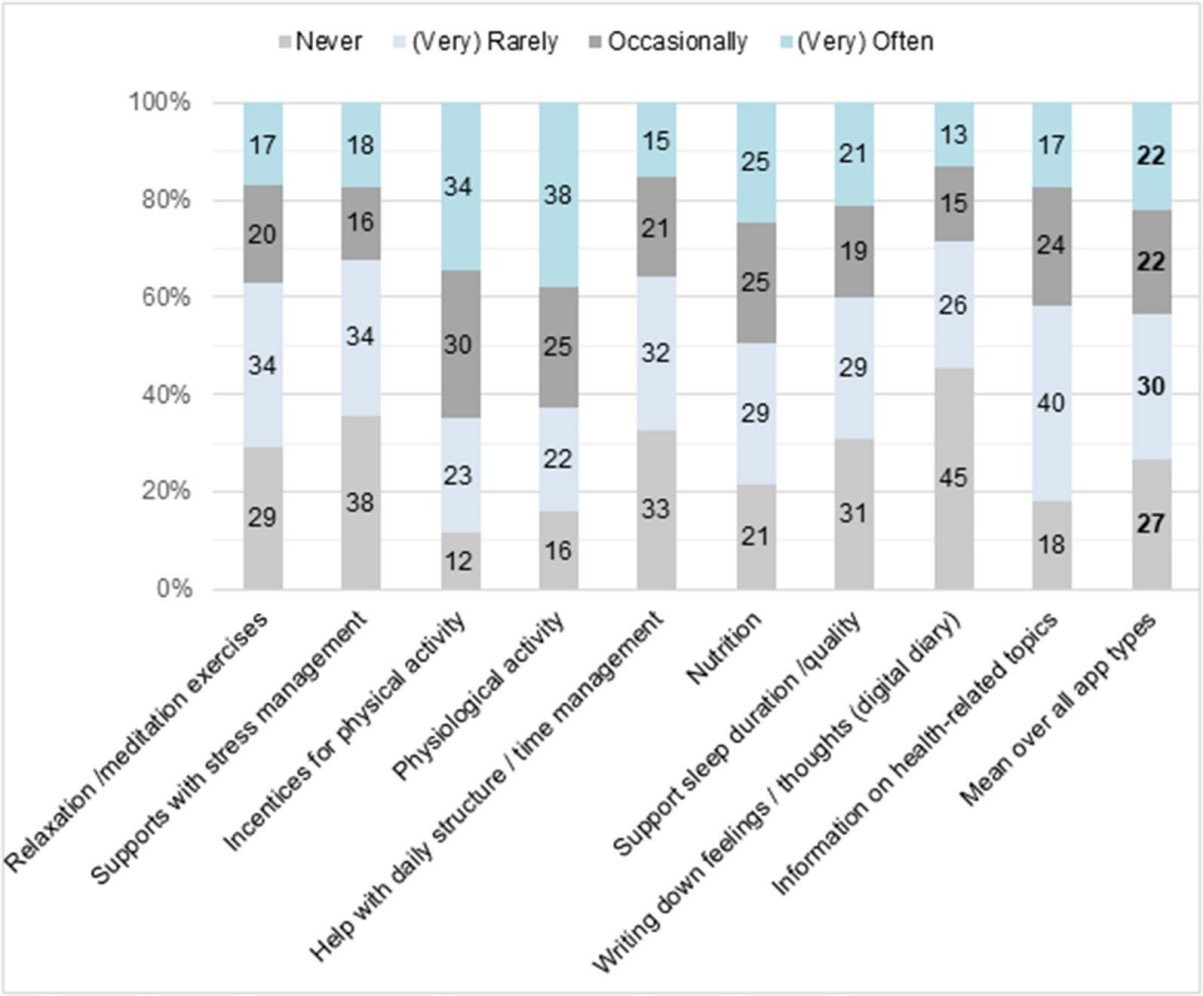
Distribution of app types used among survey participants (N = 473). The response categories “often” and “very often” were combined to “(very) often”. The response categories ‘rarely’ and ‘very rarely’ were combined to “(very) rarely”

Among survey participants who already had experiences with mHealth apps, 60% make regular use (at least once a week) of 1–2 apps, however 29% indicated to use none of their installed mHealth apps regularly. We also presented a selection of common app content or exercises to those who reported to regularly use mHealth apps (n = 473). Surprisingly, among these users, “never utilized” was selected most often (on average in 27% of cases) compared to the other answer options. Nevertheless, apps that promote physical activity or apps that track physiological data (eg, one’s heart rate) were rather common among existing app users. Less popular were apps with a diary options, apps that support stress management or relaxation/mediation apps. More details are shown in Fig. 1.

Young people in focus groups reported that they had used an mHealth app at least once (7/8) or more frequently over a short period of time (6/8), but had stopped the use of most apps. For 2 participants, this happened due to reaching their “goal”. Half of the focus group participants highlighted high costs or payment models as reasons, and for 2 participants their use ended with the free testing period of the app. In other cases, either the app was assessed to be unsuitable to reach the goal because it was too unreliable (2/8). For example, the alarm of a sleep cycle app did not go off, or the use was too demanding (3/8). For the latter, one participant stated concerning a nutrition app:

*“I don't use it very often, I only used it once or twice just to see how I eat during the day* ...*”* [P3, FG2]

*“But you did stop at some point?”* [Facilitator, CG]

*P3, FG2: “Yes, exactly, because it is very exhausting to go through this every day* …*to keep listing the products there.”*[P3, FG2]

In stressful periods (eg, final exams) 3 out of 8 focus group participants made use of mHealth apps. Two young people used mHealth apps for habit tracking (eg, to control eating habits or to self–organize).

### AI use in the context of mHealth apps

#### Young people on AI in everyday life

When asking for young peoples’ perspectives on AI in general, most participants of our focus groups were able to name central mechanisms of AI (7 of 8). In particular, the terms “learning” (5 of 8), “machine learning" [P2, FG1], and “AI as a learning algorithm” [P4, FG1] were mentioned. Participants agreed upon the principle that more data leads to better outcomes of AI, although they reported a lack of understanding of the underlying technical details:

*“I've heard of it, most definitely. Of course I don't know the technical background, but what I imagine is that if you give an input, the system learns“*[P1, FG2]

AI usage was linked to application areas such as social media (2/8), shopping (2/8), and personalized advertising (2/8). Young people also mentioned online translators (eg, google translate) or the internet of things (eg, temperature control).

Most participants had a positive attitude towards AI use in general (6/8). They reported a pragmatic approach towards AI and data usage, which implied sharing data and use mHealth apps as long as the app was useful to them. One participant elaborated:

*“I think there are two sides to this. I find it...scary, if you do not yet know exactly what is happening with your data…. On the other hand, I think it's great when I go to...Spotify ‘your mix of the week’ and I kind of like all the songs*.... [As] *long as I somehow benefit from it and know that my data is safe…, then I actually think that's good and I'm positive about it.”*[P4, FG2].

Uneasiness about the opacity of machine learning processes was mentioned rather casually (6/8) and was often associated with popular media, especially science fiction movies (eg, “Terminator” [P2, FG1]). However, both focus groups came to the conclusion that regulations and control mechanisms for AI are necessary (8/8).

#### Opportunities and risks seen by young people

Concerning the use of AI in the mHealth app we are currently developing in the living lab AI4U, as presented in focus groups, participants showed general interest and openness and expressed few to no concerns about the usage of AI (7/8). Similarly, in the online survey, only a minority (17%) reported to feel negative about the application of AI in general, and 19% negative about the embedding of AI in mHealth apps.

In focus group 1, the combination of a great variety of interventions, and the possibility to tailor the app to one’s personal needs to find “your own path” was especially desirable for one participant [P2]. Similarly, other young persons (4/8) highlighted the advantage of personalization due to the AI elements:

“*Yes, I think that in the area of mental health or health in general it could work pretty well to use such artificial intelligence, because…everyone is super individual and of course it works better if it is well adjusted to each person*.“ [FG1_S].

At this point, 5 out of 8 young people stated that they have few concerns in sharing information with the app. Participants justified broad data usage, especially if the app is useful to solve user–defined problems. Two young people argued that their smartphone already gathers many personal data, thus sharing more information was no major concern to them:

*“When I think about it, my cell phone probably already knows so much about me anyway, because it is somewhere nearby all the time...that I would maybe give more information than others.”* [P2, FG1]

**Fig. 2:**
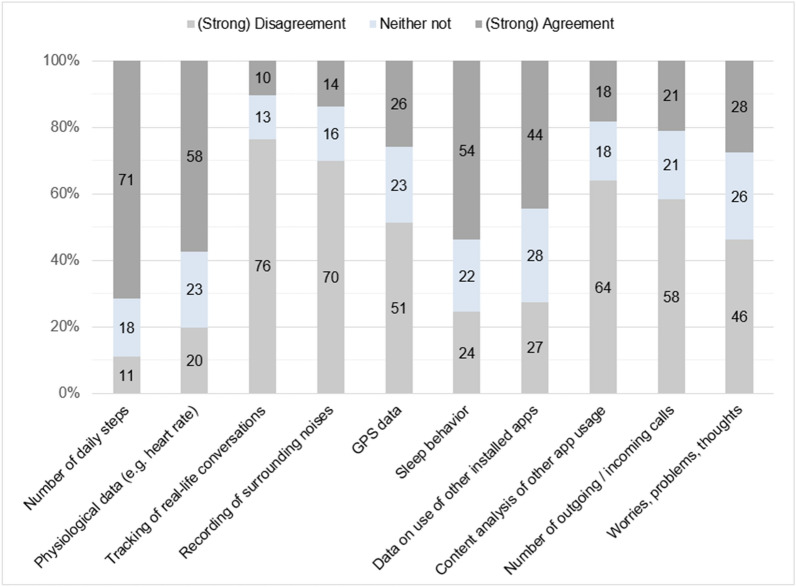
Attitudes of survey participants towards data sharing across different types of data (N = 666). The response categories “agreement” and “strong agreement” were combined to “(strong) agreement”. The response categories “strong disagreement” and “disagreement” were combined to “(strong) disagreement”

Asking which information participants were willing to provide in order to improve tailoring and functionality of AI (given all guidelines being followed), youth in the survey mostly agreed to share information like steps taken per day, physiological data (eg, heart rate), or sleep behavior, for example day/night rhythm or duration of sleep (see Fig. 2). At the same time, a majority of young persons disagreed to share real–life conversations or the recording of surrounding or ambient noises, as well as to the analysis of text or text length of messages that have been sent.

**Fig. 3:**
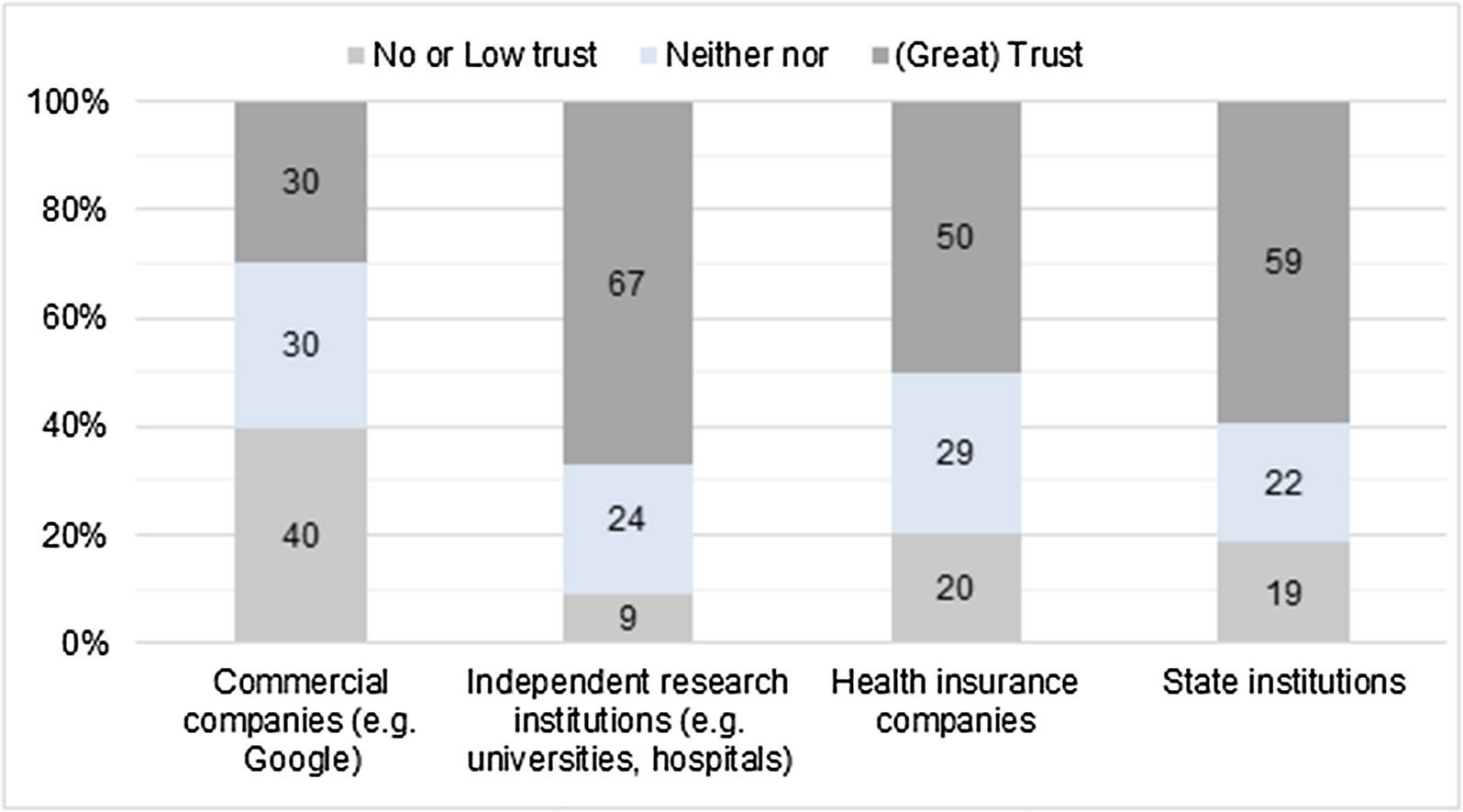
Trust in potential app developers among survey participants (N = 666). The response categories “trust” and “great trust” were combined to “(great) trust”. The response categories “no trust at all” and “low trust” were combined to “no or low trust”

During focus groups, young people also expressed reservations about AI's use of “personal” data, such as diagnoses (“app should not replace the doctor” [P2, FG2]) or one’s deeper feelings and thoughts. One male participant stated that he would share the level of stress he had but not the reason for the distress itself, like something he would put in a diary [P2, FG1]. Doubts were also expressed about the app´s functionality and ability to comprise more profound concepts (eg, melancholic feelings, thoughts about meaning in life, problems with friends or family). One male participant was skeptical about the app’s ability to grasp deeply entangled emotional problems and to give adequate answers [P4, FG1]. Reluctance to share personal information was linked to trustworthiness of the app producers (4/8), where non–commercial providers were trusted more, compared to providers with financial interests. This is in line with survey results, where only 20% deemed it very important to know who developed the app, while 23% were indifferent and 15% deemed it unimportant. Two–thirds of young people reported to have greatest trust towards independent research institutions (e.g., universities or hospitals) when compared to health insurance, state institutions, or companies such as “Google”. The latter and other companies with a potential commercial interest where trusted the least (see Fig. 3).

**Fig. 4:**
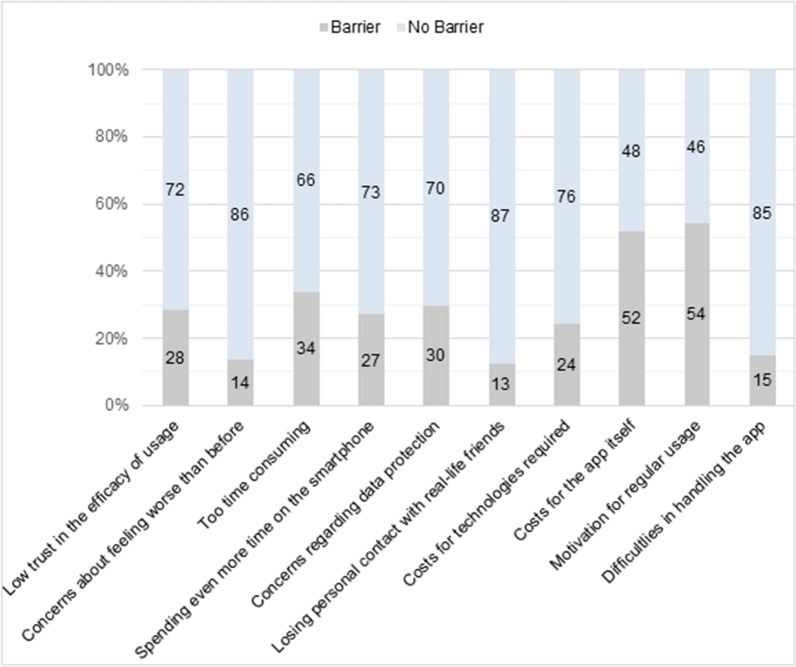
Reasons for non-usage of mHealth apps among survey participants (N = 666)

Young people in our online survey reported barriers for the use of mHealth apps including lacking motivation and costs for the app itself, while a majority assessed feeling worse than before, difficulties in handling the app or losing personal contact with real–life friends as ‘no barrier to usage’ (see Fig. 4).

#### Opportunities and risks seen by experts

Experts expressed positive views on the use of AI in mHealth apps (5/5) by emphasizing the low–threshold access to young people (2/5), especially for reaching potential risk groups early on (eg, young persons with few social contacts). Similarly positive was the experts’ view on the potential for better–informed recommendations (2/5), resulting in a high applicability of the app in counseling and school centers (3/5). However, 2 experts stressed that the app should be connected to mental health counseling facilities in order to inform users about nearby professional services [EXP1, EXP5]. Especially in cases of suicidality, the app should include further measures for risk reduction and enable access to further help for those in need (2/5). For some experts the app may serve as a supplement to face–to–face interventions or counseling services (2/5). By empowering young people to take care of their mental health themselves, the app has the potential to close existing treatment gaps in mental health promotion and prevention for youths with minor mental health problems.

From experts’ perspectives, interventions of the planned app should provide “knowledge transfer”, for example to empower young people to realize how actions, feelings and thoughts are intertwined (2/5). Further recommendations included the promotion of “self–acceptance” / “self–worth” (2/5), “physical activity” (2/5), “social skills” (2/5), and “emotion regulation”, for example getting access to one’s feelings (2/5). Young peoples’ motivation to use the mHealth app might be increased by “giving stories / everyday examples” (3/5), tailored tips and ideas for specific situations (3/5), the initiation of self–reflection (2/5) or interventions to experience self–efficacy (2/5). One expert emphasized that several youth studies underlined that communication with peers was essential for young people in internet activities, thus the option to communicate with other young people should be included in the app to ensure a continuous use [EXP3].

Experts’ concerns about the app were mostly related to not being able to reach or motivate young people for the apps’ training (3/5). Furthermore, experts underlined that various barriers for the implementation of the app need to be taken into account, such as structural obstacles, for example prejudices of school officials against mHealth apps (2/5) or persisting lack of technical equipment in schools (2/5). They reported possible negative influences in enforcing digital media overuse [EXP5] and, given the continuous pressure to self–optimize, empowering potentially problematic “perfect” self–images [EXP3]. Based on the recommendation of one psychologist, the app should therefore not arouse false expectations in young people:

*“I would see a risk if the app claimed: ‘if you go through these ten steps...then you are a different person’ (laughs).* ...*A good app would be characterized by the fact that the user does not internalize a problem centered perspective, but that he...gets the feeling: ‘I am okay’.”* [EXP5]

### Recommendations for AI–informed mHealth apps with EMA/EMIs

#### Young peoples’ recommendations in focus groups

##### App includes a variety of exercises and gives orientation

Young people underlined that the planned app should not only provide tailored exercises (to one’s needs and goals) from a broad variety of exercises, but to also give users an overview and orientation over possible “routes” (eg, training sequences), in order to support users in finding and defining “their own path” [P2, FG1]. Or as one participant stated*:*

*“...you have a lot of apps that can promote health, but you have to find the one app that suits you best ..., it’s actually exactly the other way around* [with the planned app; CG]*, so that you have an app,...that can indicate the various routes to you....That's pretty handy.” * [P2, FG1]

##### Personalized personalization–App includes users in AI decision making process

All young people demanded control over the degree of personalization through AI (8/8). One participant therefore proposed the concept of “personalized personalization” [P1, FG2], which means to be able to self–determine the time and degree of further personalization, ideally at the beginning of the app use. Also, young persons demanded flexibility and the possibility to change those settings retrospectively (2/8), for example to be able to receive suggestions for exercises once again, even after dismissing them initially (allowing for development or a change of mind).

##### App gives recommendations when “it’s smart enough”

Participants had concerns about imprecise or bad suggestions of the AI especially at the beginning of app use, due to a lack of data and therefore insufficient tailoring of the app. Thus, 2 participants proposed that AI should come into use only when sufficient personalization was reached to avoid imprecise suggestions (2/8):

*“…when I'm still at the beginning, the app doesn't know much about me yet, but it still gives me suggestions .... And then I tend to distance myself from the app…because it simply suggests things that I don't need at all. So, I think...that it would be really cool if this AI only came in at a certain point in time, when it is smart, or when it’s smart enough to step in.”*[P3, FG2]

##### App suggests solutions for difficult situations

Besides having a set of exercises at hand to volitionally practice certain aspects of mHealth, participants underlined the importance of tailored solutions (eg, a help button) in difficult moments of not feeling well (4/8).

*“*...*but that you have such a button*...wh*ere it says, no idea, ‘help me’*...*and then* ...*get the whole thing rolling.*” [P2, FG1]

For others, this could also mean to receive suggestions for activities in times of inactivity–which could be measured by passive data (eg, steps taken per day)–and for example to receive suggestions to meet up with friends (2/8).

##### App suggests distance to mobile phone

Crucial feature of the app for young people was the encouragement to refrain from the use of mobile phones (2/8). One young person especially stressed distance from social media, as precursor of stress and competitive behavior [P3, FG1].

*„ I think if an app like this gets you to turn off your cell phone and do breathing exercises, or some sport...and takes you away from it a bit, then I think it's very helpful, even though it's ‘just another app’."* [P1, FG1]

##### Mutual trainings or sharing trainings with others rarely desired

7 out of 8 participants stated at some point that they exercise for themselves and not “for others". Therefore, 5 out of 8 young people preferred not to share training sessions or outcomes with acquaintances or other app users, for example to avoid competitive behavior (3/8). Especially the public posting of achievements via the app was deemed undesirable (5/8), whereas the one–on–one exchange with close friends or family was considered acceptable by some (2/8).

##### Reasonable time requirements for app usage

Given the rationale of proper time management, participants raised concerns about the effort of app usage and time required for the personalization of the app as well as the amount of exercised the app recommends:

*„When you have to fill out super long stuff,...then it might annoy me at some point, if it is a lengthy process.“* [P3, FG2]

#### Important aspects of mHealth apps for survey participants

**Fig. 5:**
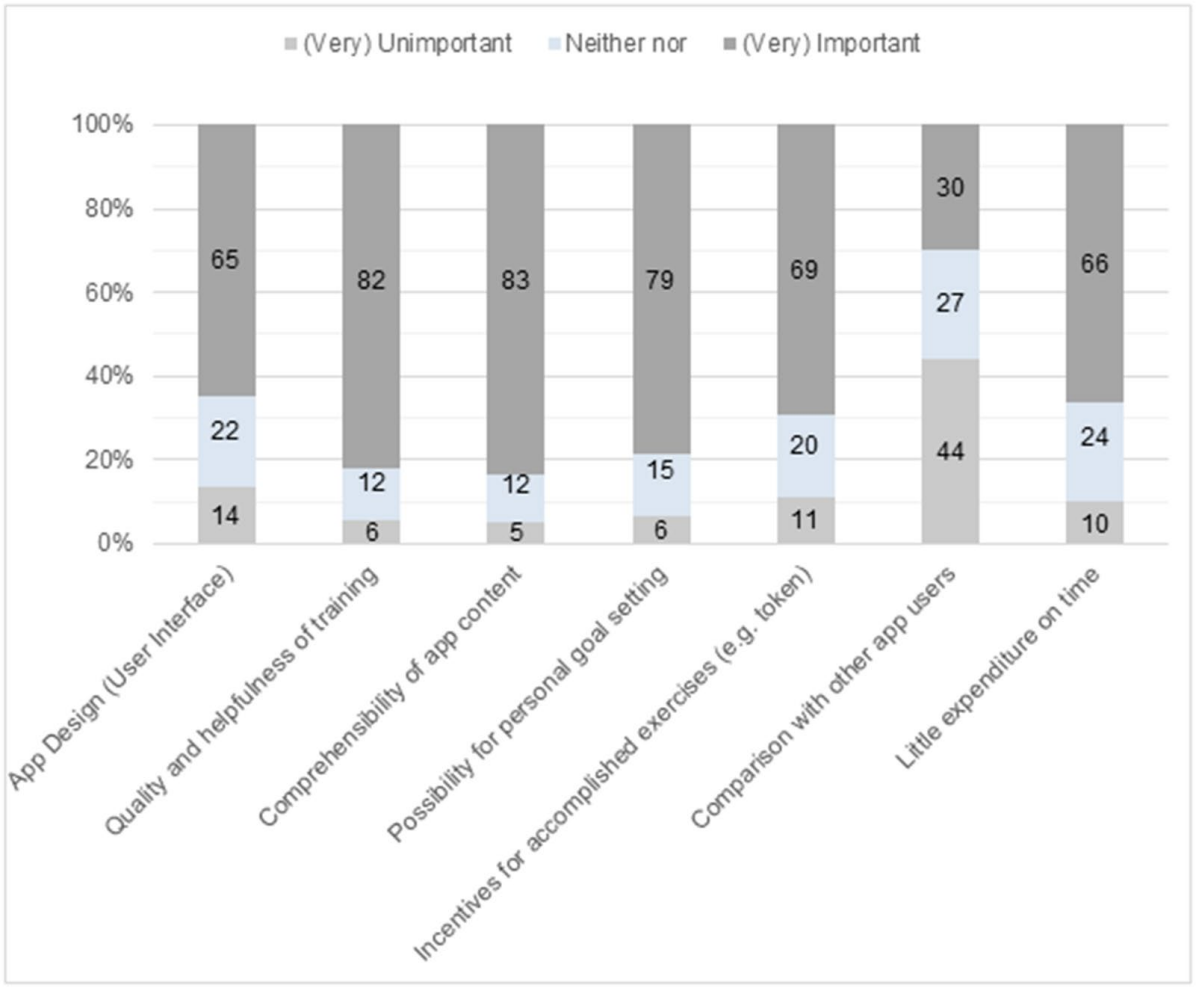
Important aspects in mHealth apps among survey participants (N = 666). The response categories “important” and “very important” were combined to “(very) important”. The response categories “very unimportant” and “unimportant” were combined to “(very) unimportant”

With respect to important aspects of the app (see Fig. 5), young people considered the “comprehensibility of app content” (84%), “quality and helpfulness of training” (82%), and the “possibility for personal goal setting” (79%) as important or very important. Compared to the other aspects, the chance for comparison with other users was deemed the least important aspect in an mHealth app.

#### Experts’ recommendations

##### Inclusion of young people into app development

Overall, experts recommended involving young people as early as possible in the app development process (5/5). This could mean to put special emphasis on “risk groups” (eg, socially disadvantaged young people, or young people displaying behavioral problems), which may have different needs concerning processes and structures of interventions (EXP4).

##### Target group–specific language

In terms of language editing, experts underlined to ensure that formal information (eg, use of data, data protection) was comprehensible. Thus not only to comply with the “clear and plain language” required by GDPR, but *also “to formulate the terms and conditions in simple language, that anyone can understand”* [EXP3]. Additionally, this should imply conveying “acceptance” or “affirmation” within texts (eg, that it takes time to perform an exercise, to practice at one's own pace) rather than taking a problem–centered approach [EXP3, EXP5].

##### Forwarding young people to counseling centers

According to experts, the app should be able to connect young people with helpful contacts in their vicinity (eg, suicide hotlines, counseling services, trust teachers) and facilitate the structured search for professional help [EXP1, EXP 5]. Thus, the app may reach risk groups at an early stage, when they are not yet likely to attend counseling or search for clinical support yet.

##### Low–threshold access to potentially endangered young people

Two experts highlighted the risk of triggering self–harming behavior or suicidality through app usage [EXP1, EXP 5], but emphasized that the planned app could also mean an early, low–threshold access to potentially endangered young people. One expert therefore recommended the careful phrasing of questions and exercises, but also stressed that suicidality and suicide ideation were often hard to recognize in counseling as well [EXP5]:

*“It’s the same as in counseling…It’s never clear how things will proceed and if we are very careful there*...*we may reach someone at a point in time when they are at risk and use the app to get them to open up somehow, to turn them around*.” [EXP5]

##### Careful framing of AI technology

Two experts raised concerns on a widespread lack of knowledge and the risk of limited acceptance of AI–informed technology. Due to false “dystopian” imaginations of AI–technology, there might be persisting reservations especially in Germany [EXP4], why an exceedingly strong focus on AI in the app could lead to reactance [EXP2]. As a counter–measure, it was stressed to lay emphasis on its benefits, for example tailored offers made possible through AI, and to guarantee as much transparency as possible [EXP2].

## Discussion

### Overview

The aim of this study was to explore attitudes, preferences and needs of young people towards AI–informed mHealth apps, together with those of crucial stakeholders in the field of mental health, to guide the further development of an app using EMA and EMIs (see Table 3). Qualitative findings concerning the individual perspectives of young people and stakeholders were complemented through survey results about young people’s current use of mHealth apps. We found that participating young people and experts were mostly open–minded towards the use of AI, as well as its implementation in an mHealth app. Results of qualitative and quantitative data are largely consistent, revealing that app content, functionality and the opportunity for personalization are the most important components for use in mHealth apps.

**Table 3:** Recommendations for AI-informed mHealth apps in qualitative research by experts and young people

**Recommendations by young people**
- App includes a variety of exercises and gives orientation
- Personalized personalization–App includes users in AI decision making process
- App gives recommendations when “it’s smart enough”
- App suggests solutions for “difficult situations”
- App suggests distance to mobile phone
- Mutual trainings or sharing trainings with others rarely desired
- Reasonable time requirements for app usage
**Recommendations by experts**
- Inclusion of young people into app development
- Target group–specific language
- Forwarding young people to counseling centers
- Low–threshold access to potentially endangered young people
- Careful framing of AI technology

### Young people’s views on AI-informed mHealth apps

In general, the results of our survey show that most young people already use mHealth apps in a broader sense (eg, to increase physical activity), and the majority can imagine using them to promote their mental health (eg, to deal with stress, reflection of one's own behavior). For our survey participants, the most important aspects seem to be quality and effectiveness of the mHealth app, followed by comprehensible content and ease of use. Personal goal setting seems to be an additional important feature of an mHealth app, which is in line with the desired app functionality, described in focus groups. In contrast, the possibility to compare one’s progress with others was deemed the least important aspect for continuous app use among participants, in both focus groups and online survey. Besides control over information sharing, also time–consuming app usage seemed to be a decisive factor that would prevent our survey participants from using mHealth apps. We see similar attitudes in focus groups, where young people raised concerns about lengthy processes of data entry for personalization or general effort of app usage. This is in line with earlier studies, where a high sampling frequency (ie, seven triggered signals a day on six consecutive days) was perceived as burdensome by young people [34] or connected to lower compliance and retention rates in participants [55]. Vachon et al. recommended in a meta–analysis of EMA studies investigating major depressive disorder, bipolar disorder, and psychotic disorder, to decrease the number of evaluations per day and use bigger time intervals between successive evaluations, in order to increase compliance in participants [55]. Other usage barriers mentioned by young people in focus groups, who had used mHealth apps before, were costs for the app itself, which lead to an end of usage after the free–trial period. The same was mentioned by survey participants. Notably, focus group participants described a problem–centered approach to the use of mHealth apps (if they used them at all), implying usage only during stressful periods (eg, exams) or when to achieve a certain predefined goal (eg, improved sleep quality). After reaching the goal, the app often had fulfilled its purpose and became irrelevant. To reach a continuous use in young people, it seems advisable not only to center the app around one “problem” or “goal”, but to keep the app flexible and adjustable to changing life situations, thus becoming a constant companion with trainings for ever–changing settings.

Although young people in our study show insecurities and a lack of (deeper) knowledge regarding the technical background of AI, they seem to be familiar with the central mechanisms or keywords (eg, terms like “machine learning” or “algorithms”). This is in line with findings of Young et al., where participants were familiar with AI in general, but not with AI in a clinical setting, and mostly linked AI to cognition (eg, in games), machines or applications (eg, robots, Google, Spotify), and to popular media (eg, movies) [35]. Young people in our study had a rather positive–pragmatic attitude towards the use of an AI–informed mHealth app. Participants’ willingness to make use of the AI–informed training was high, as long as it was useful and the potential to reach personal goals was given. Similarly, young people showed pragmatic attitudes towards data sharing: Qualitative as well as quantitative results demonstrate that the greatest commitment arises when data is provided for research (eg, universities as app providers) or optimization purposes (eg, for better tailoring and functionality of AI), rather than primarily for commercial intentions. At the same time, reasonable explanations for the required data collection and the credibility of the provider seem to be the most decisive factors when it comes to data disclosure. However, besides plausibility of data requests, positive attitudes towards data sharing turned out to decline with increasing intimacy of the data requests. In our survey as well as our focus groups, young people were not willing to give permission for analyzing text messages or tracking real life conversations, fearing data loss, the inaptitude of AI to process data of this complexity, or a general discomfort in sharing personal thoughts with anyone. Yet for some young persons this was also negotiable, as long as there were good reasons for such data queries resulting in benefits for users. Especially with regard to data security, transparency and justification for information requests seemed crucial.

### Experts’ views on AI–informed mHealth apps for young people

Similar to young people, experts have shown largely positive attitudes towards the use of AI methods and highlighted the chances of AI involvement rather than its risks. Experts emphasized that an AI–informed mHealth app has the chance to reach young people in moments when help is needed most, with measures adopted to young peoples’ specific needs. Yet experts also underlined that future mHealth apps should integrate options of forwarding young people to face–to–face support (eg, by referring to counselling services nearby), to ensure support in emergency situations. This is in line with earlier findings, in which attitudes of physicians and psychotherapists were investigated and mHealth interventions were viewed as a supplement to conventional face–to–face interventions rather than being effective on their own [56, 57]. This skepticism is also reflected in the experts' recommendation of careful formulations and contact options with respect to levels of suicide ideation [58] in cases of suicidal thoughts or self–harming behaviors, that might be triggered through questions or phrases used in the app. This demand was similarly raised by young people, given that they would like to see a “help–me” button in the app. Apart from such a button, young people did not mention suicidality or self–harm, which might be due to focus group recruitment of young people from the general population without known experiences of mental health conditions, including suicidal behavior or thoughts.

### Divergences between experts’ and young people’s views

Some divergent results were found between expert views and attitudes of young people. Firstly two experts disliked the term “mental health” due to negative connotations and semantic proximity to ‘mental illness’. They preferred the term “mental fitness” and “mental health training” in order to broaden the appeal of the planned app and to reach more young people. However, none of the young people criticized or rejected the term “mental health” in focus groups. This may reflect a change in attitudes concerning mental health in younger generations, resulting in a greater openness towards engaging in or speaking about one’s mental health or wellbeing. Further research with young people has to determine which term fits best, in order to achieve broad acceptance amongst the target group. Secondly, in contrast to young people, one expert highlighted the importance of interaction with peers via the app. From this perspective, young people mainly use the internet for communication purposes (eg, for social media, general messenger apps). However, young people in focus groups and our survey stressed that they did not want to communicate with peers in the context of an mHealth app (with few exceptions), mainly because they feared comparison, peer pressure or competition. Preferences of young people therefore point into the direction of a solitary app use. This is in line with results of Peng et al. who also pointed out that users rarely wanted to share personal information due to privacy or security concerns, or because sharing might fuse social competition [59]. Only full control with whom information was shared made sharing with small groups of individuals acceptable [59].

## Strengths and limitations

To the best of our knowledge, this mixed–method study is the first to provide an opportunity for young people and relevant stakeholders to be involved in the first step of the development process of an AI–informed mHealth app delivering EMIs. With respect to sample size, the number of participants in qualitative data collection was relatively small and due to the snowball system procedure in recruitment, the focus groups posed real–life groups. This may be the reason for a lack in controversial debates and a communication style characterized by turn–taking in answering questions. This may also be influenced by the online format that was due to first quarantine measures, where occasional problems with connectivity and overlapping audio occurred. Most participants also held or pursued a higher educational degree, which may account for better knowledge and less skepticism about technology. Future studies should include more diverse participants as well as conduct a greater number of focus groups. However, due to narrowly defined objectives and a homogeneous study population, exploratory qualitative results allowed for in–depth discussion, while the quantitative, representative data helped to form generalizability. Though a description of AI components in the planned app was given in qualitative and quantitative research, the perspectives of young people with lived experience and usage of the planned app may differ from those included in our study, which only had hypothetical scenarios to refer to. Increased experience with AI–informed mHealth tools may change young peoples’ attitudes and young people may become more comfortable with their usage and sharing of information. Experts also mostly addressed AI in the specific version discussed in the steering committee meeting (involving EMA & EMIs). Due to recruitment of experts from the steering committee and affiliation with the project, as well as the main expertise of experts in mental health promotion and prevention of young people (eg, school counseling), experts mostly followed the approach presented at the steering meeting. Expert opinions with a higher expertise in mHealth apps or AI may therefore differ. Furthermore, all experts agreed to the argumentation of the project concerning the better tailoring to the needs of young people and therefore having a lower–threshold access to youth at risk of mental health problems. Though this argument is valid due to former research of the authors [32, 34], and despite that some studies found that online help–seeking was not influenced by socioeconomic status (eg, with lower formal education) [60, 61], future research should particularly include young people with a low socioeconomic status, because their experience and perception of AI–informed mHealth apps may differ.

## Conclusions

There is a plethora of mHealth apps available in app stores that rarely use evidence–based frameworks or lack tailoring to the specific needs and interests of youth. AI–informed mHealth apps that utilize EMIs and EMA may pave the way for low–threshold interventions that allow for tailored mental health promotion and prevention with respect to person, moment, and context. Though further studies are needed to evaluate their effectiveness, our findings point towards general acceptance and interest in AI–informed mHealth apps by young persons and experts working in routine practice of mental health promotion and prevention (eg, school psychologists, social workers, educators). Results highlight a pragmatic use of mHealth apps by young people, who show openness to AI but prefer personalization of all app processes, including the chance to determine the degree of personalization itself. Also positive attitudes towards data sharing with an AI based mHealth app tended to decrease with the level of intimacy of the data requested (eg, “steps taken” were more acceptable to share than “tracking of real-life conversations”). Our findings show, that the possibility of personalization (eg, to determine one’s goals and the app’s purpose) bears high relevance for data sharing and long–term app usage in young people. To further promote a continuous use of mHealth interventions, developers should aim for user–centered approaches on mobile applications with EMIs & EMA, evaluating users’ perspectives towards AI–informed components and necessary design features that support adherence. In particular, our findings underline the necessity of collaborative research with young people and their early involvement in decision–making processes during app development. First insights from this mixed-methods approach, which poses a pre-stage of participation, will therefore be used to inform the next stages of the project, especially to implement early on participation of young people in all stages of the research process (e.g. data collection, analysis, interpretation). Considering young people’s currently poor access to mental health services and tenuous use of mHealth apps, this may guide the development of helpful applications that young people may use more regularly.

## Supplementary Information

**Additional file 1: **Interview topic guide–focus groups.

**Additional file 2: **Interview topic guide–expert interviews.

**Additional file 3: **Online survey.

**Additional file 4: **Overview of participants in the qualitative study.

**Additional file 5: **COREQ-Checklist.

**Additional file 6: **Coding Trees.

## Data Availability

The datasets obtained and analyzed during the current study are available from the corresponding author on reasonable request. Full transcripts of focus groups and expert interviews are not publicly available, as they might identify individuals, even though they are anonymized.

## Abbreviations

AI4U: Artificial Intelligence for personalized digital mental health promotion and prevention in youth
BW: Baden–Wuerttemberg
EMA: Ecological momentary assessment
EMI: Ecological momentary intervention
GDPR: General data protection regulation
RNN: Recurrent neural networks
mHealth: Mobile mental health
mHealth apps: Mobile mental health apps

## References

[1] Gore FM, Bloem PJN, Patton GC, Ferguson J, Joseph V, Coffey C (2011). Global burden of disease in young people aged 10–24 years: a systematic analysis. The Lancet.

[2] Kessler RC, Berglund P, Demler O, Jin R, Merikangas KR, Walters EE (2005). Lifetime prevalence and age-of-onset distributions of DSM-IV disorders in the national comorbidity survey replication. Arch Gen psychiatry.

[3] WHO. Prevention of mental disorders. Effective interventions and policy options. Geneva; 2004.

[4] McGorry P, Bates T, Birchwood M (2013). Designing youth mental health services for the 21st century: examples from Australia, Ireland and the UK. Br J Psychiatry Suppl.

[5] Das JK, Salam RA, Lassi ZS, Khan MN, Mahmood W, Patel V, Bhutta ZA (2016). Interventions for adolescent mental health: an overview of systematic reviews. J Adolesc Health.

[6] Colizzi M, Lasalvia A, Ruggeri M (2020). Prevention and early intervention in youth mental health: is it time for a multidisciplinary and trans-diagnostic model for care?. Int J Ment Health Syst.

[7] Hollis C, Falconer CJ, Martin JL, Whittington C, Stockton S, Glazebrook C, Davies EB (2017). Annual research review: digital health interventions for children and young people with mental health problems - a systematic and meta-review. J Child Psychol Psychiatry.

[8] Clarke AM, Kuosmanen T, Barry MM (2015). A systematic review of online youth mental health promotion and prevention interventions. J Youth Adolesc.

[9] Ebert DD, Cuijpers P, Muñoz RF, Baumeister H (2017). Prevention of mental health disorders using internet- and mobile-based interventions: a narrative review and recommendations for future research. Front Psychiatry.

[10] Ravens-Sieberer U, Kaman A, Erhart M, Devine J, Schlack R, Otto C (2021). Impact of the COVID-19 pandemic on quality of life and mental health in children and adolescents in Germany. Eur Child Adolesc Psychiatry.

[11] Marques de Miranda D, Da Silva AB, Sena Oliveira AC, Simoes-E-Silva AC (2020). How is COVID-19 pandemic impacting mental health of children and adolescents?. Int J Disaster Risk Reduct.

[12] Pierce M, Hope H, Ford T, Hatch S, Hotopf M, John A (2020). Mental health before and during the COVID-19 pandemic: a longitudinal probability sample survey of the UK population. Lancet Psychiatry.

[13] Rauschenberg C, Schick A, Goetzl C, Roehr S, Riedel-Heller SG, Koppe G (2021). Social isolation, mental health, and use of digital interventions in youth during the COVID-19 pandemic: a nationally representative survey. Eur Psychiatry.

[14] Klasnja P, Pratt W (2012). Healthcare in the pocket: mapping the space of mobile-phone health interventions. J Biomed Inform.

[15] Malla A, Iyer S, McGorry P, Cannon M, Coughlan H, Singh S (2016). From early intervention in psychosis to youth mental health reform: a review of the evolution and transformation of mental health services for young people. Soc Psychiatry Psychiatr Epidemiol.

[16] Marshall JM, Dunstan DA, Bartik W (2020). Apps with maps-anxiety and depression mobile apps with evidence-based frameworks: systematic search of major app stores. JMIR Mental Health.

[17] Donker T, Petrie K, Proudfoot J, Clarke J, Birch M-R, Christensen H (2013). Smartphones for smarter delivery of mental health programs: a systematic review. J Med Internet Res.

[18] Grist R, Porter J, Stallard P (2017). Mental health mobile apps for preadolescents and adolescents: a systematic review. J Med Internet Res.

[19] Larsen ME, Huckvale K, Nicholas J, Torous J, Birrell L, Li E, Reda B (2019). Using science to sell apps: evaluation of mental health app store quality claims. NPJ Digit Med.

[20] Bergin AD, Vallejos EP, Davies EB, Daley D, Ford T, Harold G (2020). Preventive digital mental health interventions for children and young people: a review of the design and reporting of research. NPJ Digit Med.

[21] Michel T, Tachtler F, Slovak P, Fitzpatrick G (2019). A review of youth mental health promotion apps towards their fit with youth media preferences. EAI Endorsed Trans Pervasive Health Technol.

[22] Lecomte T, Potvin S, Corbière M, Guay S, Samson C, Cloutier B (2020). Mobile apps for mental health issues: meta-review of meta-analyses. JMIR Mhealth Uhealth.

[23] Kenny R, Dooley B, Fitzgerald A (2016). Ecological momentary assessment of adolescent problems, coping efficacy, and mood states using a mobile phone app: an exploratory study. JMIR mental health.

[24] Stone A, Shiffman S, Atienza A, Nebeling L (2007). The science of real-time data capture: self-reports in health research.

[25] Heron KE, Smyth JM (2010). Ecological momentary interventions: incorporating mobile technology into psychosocial and health behaviour treatments. Br J Health Psychol.

[26] Reininghaus U (2018). Ambulatorische interventionen in der psychiatrie: das Momentum für Veränderung im alltäglichen sozialen kontext ecological momentary interventions in psychiatry the momentum for change in daily social context. Psychiatr Prax.

[27] Myin-Germeys I, Klippel A, Steinhart H, Reininghaus U (2016). Ecological momentary interventions in psychiatry. Curr Opin Psychiatry.

[28] Suhara Y, Xu Y, Pentland A'. DeepMood: Forecasting Depressed Mood Based on Self-Reported Histories via Recurrent Neural Networks. In: Barrett R, Cummings R, Agichtein E, Gabrilovich E, editors. WWW '17: 26th International World Wide Web Conference; 03 04 2017 07 04 2017; Perth Australia. Republic and Canton of Geneva, Switzerland: International World Wide Web Conferences Steering Committee 04032017. 715–724 10.1145/3038912.3052676.

[29] Koppe G, Guloksuz S, Reininghaus U, Durstewitz D (2019). Recurrent neural networks in mobile sampling and intervention. Schizophr Bull.

[30] Durstewitz D, Koppe G, Meyer-Lindenberg A (2019). Deep neural networks in psychiatry. Mol Psychiatry.

[31] Daemen M, Postma MR, Lindauer R, Hoes-van der Meulen I, Nieman D, Delespaul P (2021). Efficacy of a transdiagnostic ecological momentary intervention for improving self-esteem (SELFIE) in youth exposed to childhood adversity: study protocol for a multi-center randomized controlled trial. Trials.

[32] Reininghaus U, Depp CA, Myin-Germeys I (2016). Ecological Interventionist causal models in psychosis: targeting psychological mechanisms in daily life. Schizophr Bull.

[33] Reininghaus U, Klippel A, Steinhart H, Vaessen T, van Nierop M, Viechtbauer W (2019). Efficacy of acceptance and commitment therapy in daily life (ACT-DL) in early psychosis: study protocol for a multi-centre randomized controlled trial. Trials.

[34] Rauschenberg C, Boecking B, Paetzold I, Schruers K, Schick A, van Amelsvoort T, Reininghaus U (2021). A compassion-focused ecological momentary intervention for enhancing resilience in help-seeking youth: uncontrolled pilot study. JMIR Mental Health.

[35] Young AT, Amara D, Bhattacharya A, Wei ML (2021). Patient and general public attitudes towards clinical artificial intelligence: a mixed methods systematic review. Lancet Digital Health.

[36] Balaskas A, Schueller SM, Cox AL, Doherty G (2021). Ecological momentary interventions for mental health: a scoping review. PLoS ONE.

[37] McCradden MD, Sarker T, Paprica PA (2020). Conditionally positive: a qualitative study of public perceptions about using health data for artificial intelligence research. BMJ Open.

[38] Coughlan H, Cannon M, Shiers D, Power P, Barry C, Bates T (2013). Towards a new paradigm of care: the International declaration on youth mental health. Early Interv Psychiatry.

[39] Kendal SE, Milnes L, Welsby H, Pryjmachuk S (2017). Prioritizing young people's emotional health support needs via participatory research. J Psychiatr Ment Health Nurs.

[40] Larsson I, Staland-Nyman C, Svedberg P, Nygren JM, Carlsson I-M (2018). Children and young people's participation in developing interventions in health and well-being: a scoping review. BMC Health Serv Res.

[41] Garrido S, Cheers D, Boydell K, Nguyen QV, Schubert E, Dunne L, Meade T (2019). Young people's response to six smartphone apps for anxiety and depression: focus group study. JMIR Mental Health.

[42] Mayer G, Gronewold N, Alvarez S, Bruns B, Hilbel T, Schultz J-H (2019). Acceptance and expectations of medical experts, students, and patients toward electronic mental health apps: cross-sectional quantitative and qualitative survey study. JMIR Mental Health.

[43] Orlowski S, Lawn S, Matthews B, Venning A, Wyld K, Jones G (2016). The promise and the reality: a mental health workforce perspective on technology-enhanced youth mental health service delivery. BMC Health Serv Res.

[44] Hanson WE, Creswell JW, Clark VLP, Petska KS, Creswell JD (2005). Mixed methods research designs in counseling psychology. J Couns Psychol.

[45] Creswell JW, Plano Clark VL (2018). Designing and conducting mixed methods research.

[46] Kuckartz U (2014). Mixed Methods.

[47] Rauschenberg C, Goetzl C, Schick A, Koppe G, Durstewitz D, Krumm S, Reininghaus U (2021). Living lab AI4U—artificial intelligence for personalized digital mental health promotion and prevention in youth. Eur J Pub Health.

[48] Federal Ministry of Science, Education, and Culture of Baden-Wuerttemberg. Baden-Württemberg fördert Reallabore. 2020. https://mwk.baden-wuerttemberg.de/de/forschung/forschungspolitik/wissenschaft-fuer-nachhaltigkeit/reallabore/. Accessed 20 Jan 2022.

[49] Federal Ministry of Science, Education, and Culture of Baden-Wuerttemberg. Wissenschaftsministerium fördert zwei Reallabore Künstliche Intelligenz mit insgesamt rund 1,6 Mio. Euro: Pressemitteilung Nr. 151/2020. 2020. https://mwk.baden-wuerttemberg.de/de/service/presse-und-oeffentlichkeitsarbeit/pressemitteilung/pid/wissenschaftsministerium-foerdert-zwei-reallabore-kuenstliche-intelligenz-mit-insgesamt-rund-16-mio. Accessed 20 Jan 2022.

[50] Tong A, Sainsbury P, Craig J (2007). Consolidated criteria for reporting qualitative research (COREQ): a 32-item checklist for interviews and focus groups. Int J Qual Health Care.

[51] Kuckartz U (2018). Qualitative inhaltsanalyse: methoden, praxis, computerunterstützung.

[52] Kuckartz U (2014). Qualitative text analysis: a guide to methods, practice & using software.

[53] Rieger A, Gaines A, Barnett I, Baldassano CF, Connolly Gibbons MB, Crits-Christoph P (2019). Psychiatry outpatients' willingness to share social media posts and smartphone data for research and clinical purposes: survey study. JMIR Form Res.

[54] Torous J, Chan SR, Yee-Marie Tan S, Behrens J, Mathew I, Conrad EJ (2014). Patient smartphone ownership and interest in mobile apps to monitor symptoms of mental health conditions: a survey in four geographically distinct psychiatric clinics. JMIR Mental Health.

[55] Vachon H, Viechtbauer W, Rintala A, Myin-Germeys I (2019). Compliance and retention with the experience sampling method over the continuum of severe mental disorders: meta-analysis and recommendations. J Med Internet Res.

[56] Schröder J, Berger T, Meyer B, Lutz W, Hautzinger M, Späth C (2017). Attitudes towards internet interventions among psychotherapists and individuals with mild to moderate depression symptoms. Cogn Ther Res.

[57] Tonn P, Reuter SC, Kuchler I, Reinke B, Hinkelmann L, Stöckigt S (2017). Development of a questionnaire to measure the attitudes of laypeople, physicians, and psychotherapists toward telemedicine in mental health. JMIR Mental Health.

[58] Sander L, Gerhardinger K, Bailey E, Robinson J, Lin J, Cuijpers P, Mühlmann C (2020). Suicide risk management in research on internet-based interventions for depression: a synthesis of the current state and recommendations for future research. J Affect Disord.

[59] Peng W, Kanthawala S, Yuan S, Hussain SA (2016). A qualitative study of user perceptions of mobile health apps. BMC Public Health.

[60] Pretorius C, Chambers D, Coyle D (2019). Young people's online help-seeking and mental health difficulties: systematic narrative review. J Med Internet Res.

[61] Best P, Manktelow R, Taylor BJ (2016). Social work and social media: online help-seeking and the mental well-being of adolescent males. Br J Soc Work.

