# Supplementary material for: Artificial intelligence-informed mobile mental health apps for young people: a mixed-methods approach on users’ and stakeholders’ perspectives
Source: Child Adolesc Psychiatry Ment Health. 2022 Nov 17;16:86. doi: 10.1186/s13034-022-00522-6 (PMC9672578; doi:10.1186/s13034-022-00522-6)
Supplement: Supplementary file 1 — Additional file 1: Interview topic guide–focus groups. [file 13034_2022_522_MOESM1_ESM.pdf]

Multimedia Appendix 1

## Interview Topic Guide – Focus Groups[1]

### I. Opening:

Explanations about the interest and goal of the focus group

Short introductory round: name, age, type of school / degree (if obtained)

### II. Basics / acceptance of mental health promotion through apps

What does “mental health” mean to you?

What were the issues / situations for you (in the last 12 months) that reduced your mental well-being / had a negative impact on mental health? Where you were not doing „not so well“?

What are you already doing to promote / strengthen your mental health (sport, meditation, etc.)? Which offers do you know?

| |
|---|
| **INPUT**: Brief explanation of what we mean by “mental health”, “health promotion” and “health promoting apps”. (3-4 Sentences) |

After this brief explanation of mental health: What are you already doing to increase your “well-being” / your “emotional resilience”?

Do you know “health promoting apps”? If yes, which? On which devices do you use them? (Smartwatch, VR, etc.)

Do you use health promoting apps or have you already used apps? If yes, which?

Probe:

... for example meditation app (like Calm)

... for example sleep measurement apps

If so, why do you use this app or did you use this app? What is / was good about it and what is bad?

How long have you been using this app or have you used this app (days, weeks & how often a day)?

What are the functions of these apps?
(eg, general information, sending reminders for training / relaxation, are sensors used (eg, GPS), Motivation messages, personalized exercises, etc.)

If not, why don't you use such apps? (eg, Concerns about data protection, too exhausting, etc.)

Would you be willing to use an app to promote your mental health in the future?

[1] Focus groups were conducted in German. The topic guide was later translated to English.

What do you want with such an app? What would be good and what would be bad?

What kind of exercises / training could you imagine doing?

Probe: ... for example meditation training
... for example answering queries about mood

Where do you see difficulties / problems? (eg, usage, data protection, etc.)

Could you imagine sharing results with others? (eg, family, friends, in a forum (with strangers), with the doctor treating you, etc.)

Could you imagine doing exercises together with others? (eg, Meditation with friends, etc. or rather alone !?)

Do you think there are differences in app usage among men / women and girls / boys? V.a. Regarding Mental Health Promotion Apps?

How important is it to you that the app is tailored specifically to you, i.e. personalized?

## III. Basic information on artificial intelligence:

Have you heard of AI? What does this term mean?

Where do you think that AI is already being used in everyday life?

What do you think about the use of AI?

## IV. The use of AI methods in health apps

**INPUT**: Short explanation (with 4-6 Powerpoint-Slides) of the used AI and how it can promote mental health.

What do you think of the idea of using AI in the field of health apps? Does using artificial intelligence to personalize apps for mental health promotion make sense to you?

Do you have any concerns?

Which data would you enter into the AI in order to receive personalized offers (eg, exercises that are specially tailored to you)?

Give examples: body size, high school diploma, previous illnesses, eating habits, sleep rhythm, exact location, etc.

## V. Participation in the creation of an app with EMI / EMAs

Could you imagine participating on such a health-promoting app for young people?

If so, what could your participation look like? / What do you want to work on?

Do you want to create questions for the app and being involved in the wording (eg, for explaining "mental health" or "well-being")

Could you imagine being the first ones to test and give feedback on the app?

Where do you think it is especially important to include young people in app development? (eg, in which function (as researchers?); in which work processes (designing the interface of the app, etc.))

## VI. Final Questions

Is there anything else on your part that has not yet been discussed in the interview, but that is important to you (on the subject of AI, health promotion)?
