# Supplementary material for: Artificial intelligence-informed mobile mental health apps for young people: a mixed-methods approach on users’ and stakeholders’ perspectives
Source: Child Adolesc Psychiatry Ment Health. 2022 Nov 17;16:86. doi: 10.1186/s13034-022-00522-6 (PMC9672578; doi:10.1186/s13034-022-00522-6)
Supplement: Supplementary file 2 — Additional file 2: Interview topic guide–expert interviews. [file 13034_2022_522_MOESM2_ESM.pdf]

Multimedia Appendix 2

Interview Topic Guide – Expert Interviews[1]

I. Opening

Thank you Mr./Mrs. [XX], for agreeing to be interviewed as an expert in the "Reallabor" Artificial Intelligence (AI) Mannheim.

We have already discussed the contents of the project in detail in the steering committee. In the following interview, we would like to learn more about the role the promotion of mental health in young people already plays in your professional context and what role a health app could play in the future. Often this means vague questions, where we would be interested in ideas that come to you.

To begin with, we wanted to emphasize that the app is meant for young people in general not specifically for those with previous mental illnesses. With the help of artificial intelligence, we want to provide a personalized mMhealth app to promote the mental health of young people. In other words, an app that learns and adapts to the individual and proposes exercises or interventions, for example to reduce stress, for relaxation in general or to reflect on moods.

But first of all, we would like to ask you to describe your position and briefly describe your field of work.

II. Mental health promotion in the professional context of the expert

What does “mental health” mean to you? And what does promotion of mental health mean?

> [Possibly: Brief explanation of the "broad" term of “mental health promotion” in the Reallabor: “We have a broad definition of mental health and mental health promotion, which f.e. also includes sports activities or exercises. Ergo it means a wide range of exercises and interventions within the scope of the app.”]

What role does the promotion of mental health in young people play in your work?

How does health promotion (of young people) currently look like in your professional context? Do you know similar / concrete programs (from colleagues or others)?

Where are the problems / barriers in mental health promotion? (for measures, processes, etc.). What do parents, students, teachers and school administrators say about this? What are / would be beneficial factors?

III. Relevance of apps in health promotion

What role do **health-promoting apps** already play in your job? What do parents, students, teachers and school administrators say about this?

How / where can health apps be useful from your point of view?

Where is there a need to use these apps (eg, on which topics (such as fitness, mental health, etc.), competencies (mindfulness, etc.))?

[1] Expert interviews were conducted in German. The topic guide was later translated to English.

Can you give examples of questions from your practice that you think would be important to ask in such an app? (eg, questions about self-harming behavior or similar?)

What could such an app look like? Do you already know a good example of a similar app? In your opinion, why did this one succeed?

What must be taken into account when introducing such an app to young people?

Should peers or adults perform, for example the introduction / training of the app?

Should the young people be addressed as a group or rather individually in interventions?

IV. Opportunities, Limits, and Concerns

What would you expect from a “good” app for mMhealth promotion among young people? What should it do? How could quality be measured?

Where do you see the limit of such apps?

Do you have concerns about the use of AI in the context of promoting health among young people? If so, what type are they?

Where could be risks in health promotion? (eg, suicidal teens)

What could be measures to minimize that risk?

V. Opportunities of the participation of experts / practitioners

Where do you think the participation of experts / practitioners is important when creating the app? How could such a participation look like?

On what components would you like to work on / support our work?

VI. Final Questions

If you imagine health promotion in 5 years, what opportunities for development do you see here?

Is there anything that has not been discussed in the interview, but is important to you?
