# Supplementary material for: Artificial intelligence-informed mobile mental health apps for young people: a mixed-methods approach on users’ and stakeholders’ perspectives
Source: Child Adolesc Psychiatry Ment Health. 2022 Nov 17;16:86. doi: 10.1186/s13034-022-00522-6 (PMC9672578; doi:10.1186/s13034-022-00522-6)
Supplement: Supplementary file 3 — Additional file 3: Online survey. [file 13034_2022_522_MOESM3_ESM.pdf]

Multimedia Appendix 3

Online Survey[1]

I. Declaration of consent

0. Do you agree to participate in this online survey?
   - o Yes
   - o No

II. Sociodemographics

1. How old are you?
   ______________________ (years)

2. What is your gender?
   - o Diverse
   - o male
   - o female

3. What is your main occupation at the moment?
   - o I am a Pupil
   - o I am doing an apprenticeship/further education
   - o I am a student
   - o I am employed
   - o I am self-employed
   - o I am a volunteer/doing a free social year (FSJ)
   - o I am a househusband/housewife/on parental leave/on maternity leave
   - o I am in early retirement/disabled
   - o I am unemployed or looking for a job
   - o I am waiting for a place at university/taking a year off
   - o Other (please specify): ______

3.1 Which type of school do you attend?
   - o Hauptschule (Secondary general school for grades 5 through 9 or 10)
   - o Realschule (more practical secondary school for grades 5 through 10)
   - o Gesamtschule (integrated comprehensive school for grades 5 through 12 or 13)
   - o Technical college
   - o Gymnasium (more academic secondary school for grades 5 through 12 or 13)
   - o Other type of school not mentioned: ______

3.2 What is your highest level of education/degree?
   - o Hauptschulabschluss (general education school leaving certificate after grade 9)
   - o Mittlerer Bildungsabschluss (general education school leaving certificate after grade 10)
   - o Fachabitur (technical college entrance qualification)
   - o High School / General qualification for university)

[1] The online survey was conducted in German. The questionnaire was later translated to English.

- Completed vocational training
- Completed studies (bachelor's, master's, master's, state examination, diploma)
- Doctoral degree
- No degree
- I don't know/I don't want to give any information

4. What is your parents' highest level of education/degree?
   (Grid Scale: Mother / Father)

- Hauptschulabschluss (general education school leaving certificate after grade 9)
- Mittlerer Bildungsabschluss (general education school leaving certificate after grade 10)
- Fachabitur (technical college entrance qualification)
- High School / General qualification for university)
- Completed vocational training
- Completed studies (bachelor's, master's, master's, state examination, diploma)
- Doctoral degree
- No degree
- I don't know/I don't want to give any information

5. Were you born in Germany?
   - Yes
   - No

5.1 In which country were you born?
- Turkey
- Poland
- Russia
- Romania
- Italy
- Greece
- Kazakhstan
- Syria
- Other (please specify): ______

6. Were your parents (mother and father) born in Germany?
   - Yes
   - No

6.1 In which country/countries was your mother or father born?
(Grid Scale: Mother / Father)

- Germany
- Turkey
- Poland
- Russia
- Romania
- Italy
- Greece
- Kazakhstan
- Syria

- Other (please specify): ______

7. Were your grandparents (grandmother and grandfather) born in Germany?
    - Yes
    - No

7.1 In which country/countries were they born?
(Grid Scale: Grandmother / Grandfather)

- Germany
- Turkey
- Poland
- Russia
- Romania
- Italy
- Greece
- Kazakhstan
- Syria
- Other (please specify): ______

8. Who do you live with?
    - Parents/family
    - Friends (shared flat)
    - Acquaintances/unknowns (shared flat)
    - Other family members, not parents/siblings (eg, grandparents)
    - Partner/spouse without child/children
    - Partner/spouse with child/children
    - Alone with a child/children
    - Alone
    - Other (please specify): ______

8.1 How many people live with you?
- Another person
- Two more people
- Three more people
- Four more people
- More than 4 other people

9. You are...
    - ...in a relationship/married
    - ...single/single
    - …divorced
    - ...not certain/unexplained

III. Technology Use and Ownership

10. How often do you use the following technologies?
(Grid Scale: Never … Very often)

    - Smartphone
    - Smartwatch or fitness bracelet/tracker
    - Virtual Reality Headset
    - Augmented/augmented reality glasses (excluding smartphones with this function)

11. Is there a specific reason why you have never used one or more of these technologies? (Selection of several options possible)

    - Privacy concerns
    - Too expensive
    - No interest
    - Negative consequences of using one or more of these technologies (eg, feelings, concentration)
    - I am generally sceptical about the use of technology
    - Technologies are not really available yet
    - I don't have time to consider using these technologies
    - Other (please specify): ______

IV. Use of technologies for health promotion and monitoring

12. Would you use an app to...
(Grid Scale: Strongly disagree … Strongly agree)

    - ...deal better with everyday stress (eg, exam stress)?
    - ...deal better with worries or fears in everyday life?
    - ...be more physically active (eg, do more sports)?
    - ...learn more about your behavior/habits (eg, sleep, nutrition, exercise)?
    - ....to feel better in general?
    - ...be more aware of your use of social media (eg, Facebook, WhatsApp, Instagram, Snapchat) or computer games?
    - ...deal better with worries or fears in everyday life?

13. Infopage: We speak of "health apps" when technologies (eg, smartphones) are used for the areas mentioned above. Health apps are apps that support you in everyday life to deal better with stress or worries, to be more physically active or to improve certain habits/behaviors (eg, quality of sleep, exercise).

14. Is there an interest in your circle of friends to use mHealth apps?
    - Strongly disagree
    - Disagree
    - Neither nor
    - Agree
    - Strongly agree

15. Could you imagine using health apps using the following technologies?
(Grid Scale: Strongly disagree…Strongly agree)

- Smartphone
- Smartwatch or fitness bracelet/tracker
- Virtual reality headset
- Augmented reality glasses (excluding smartphones with this function)

16. Could you imagine using health apps that use several technologies at once to offer you their help/content (eg, a stress management training or sports program that is offered on the smartphone and a virtual reality headset and builds on each other)?

- Strongly disagree
- Disagree
- Neither nor
- Agree
- Strongly agree

17. How would you prefer to use mHealth apps? (Selection of several options possible)
- Alone, without another person
- Together with other acquaintances/friends
- Along with other users/unknowns
- Together with a digital assistant/trainer (eg, chatbot)
- I am generally not interested in using mHealth apps

18. How important would the following aspects be for you in a health app:
(Grid Scale: Very important…very unimportant)

- Appearance of the app (user interface/design)
- Quality and effectiveness of the content
- Ease of use/comprehensibility of content
- Possibility to set goals (eg, daily/weekly goals)
- Incentives/gamification within the app (eg, levels, scores, motivating reminders)
- Possibility to compare progress with others
- As little time intensive as possible
- Other (please specify): ______

19. What reasons would prevent you from using health apps?
(Selection of several options possible)

- I don't think it helps me
- I'm concerned that afterwards I'll feel worse than before
- It would be too exhausting / time consuming for me
- I don't want to spend even more time using technologies
- I have concerns about data protection
- I am concerned that personal contact with other people will be lost
- None of the reasons mentioned

V. MHealth app usage

20. Do you already use health apps (eg, for relaxation or to increase physical activity)?
    - Never
    - Very rarely
    - Rarely
    - Occasionally
    - Often
    - Very often

20.1 How many mHealth apps do you currently have on your smartphone?
- 1-2
- 3-4
- 5-6
- more than 6

20.2 How many health apps do you use regularly (at least once a week)?
- 1-2
- 3-4
- 5-6
- more than 6

20.3 Please state the name of the app/s that you currently have installed and use regularly:

____

20.4 Based on all health apps you have used so far, how often have you used the following offers of help/content?
(Carousel: Never…Very often)
- Relaxation, meditation and mindfulness exercises
- Support in stress management
- Prompts to be physically more active (sports)
- Measurement of physiological/physical activity (heart rate, pedometer)
- Help with daily structure/time management
- Help with nutrition / diet
- Opportunity to share health information with others (online forums)
- Support for sleep duration or sleep quality
- Write down feelings, thoughts or activities (digital diary)
- Information about health-related topics

20.5 What help/content was offered that has not yet been mentioned?

____

20.6 Related to the app you used most: How often have you used the app approximately?

- Installed but never used
- 1-2 times
- 3-10 times
- 11-50 times
- More than 50 times

20.7 What motivated you to use this app over a longer period of time? (Selection of several options possible)

- App appearance
- Quality, scope and effectiveness of the content offered
- ease of use
- Opportunities to tailor the app to your needs and preferences/interests
- Possibility to define your own goals
- Incentives within the app to do more exercises
- Opportunity to compare yourself to others
- Other (please specify): ______

20.8 Related to the app you used most: Did using this app help you?

- Strongly disagree
- Disagree
- Neither nor
- Agree
- Strongly agree

20.9 Related to the app you used most: would you recommend the app to friends or acquaintances?

- Strongly disagree
- Disagree
- Neither nor
- Agree
- Strongly agree

21. Are you generally interested in using health apps?

- Strongly disagree
- Disagree
- Neither nor
- Agree
- Strongly agree

22. Welche Hilfsangebote oder Inhalte würden Dir am ehesten zusagen und auf Dein Interesse stoßen? (Carousel: Strongly disagree…Strongly aggree)

- Relaxation, meditation and mindfulness exercises
- Support in stress management
- Prompts to be physically more active (sports)
- Measurement of physiological/physical activity (heart rate, pedometer)
- Help with daily structure/time management

- Help with nutrition / diet
- Opportunity to share health information with others (online forums)
- Support for sleep duration or sleep quality
- Write down feelings, thoughts or activities (digital diary)
- Information about health-related topics

23. In general, what difficulties / barriers do you see when using mHealth apps? (Selection of several options possible)

- Motivation / interest to use the app over a longer period of time
- Difficulty using the app
- Costs for technologies required (purchase of the smartphone)
- Cost of the app
- The opinion that health apps don't help anyway
- Technical implementation and / or difficulties
- Privacy concerns
- Other (please specify): ______

VI. AI usage to personalize intervention components

24. Infopage: Every day we use so-called artificial intelligence, or AI for short. Mostly without us noticing. When we search for videos on YouTube, for music on Spotify, for information on Google, or when we use Siri and Alexa, AI always plays an important role. Just like us humans, AI systems are able to make decisions based on information. Similar to us, AI systems can learn over time. For this, the AI needs as much information as possible. The more information an AI receives, the better it can solve the tasks and problems assigned to it and make decisions.

25. In general, how do you feel about the use / application of artificial intelligence?
    - Very negative
    - Negative
    - Neither nor
    - Positive
    - Very positive

26. Through the application of artificial intelligence, the offers of help/content shown to you in a health app could also be adapted to your preferences/interests. What do you think about the use of artificial intelligence in mHealth apps?
    - Very negative
    - Negative
    - Neither nor
    - Positive
    - Very positive

27. In order to adapt the health app to you using artificial intelligence, as much information/data as possible is required from you. Would you be willing to provide the following information / data to improve the AI, provided that all data protection guidelines are complied with? (Grid Scale: Strongly disagree…Strongly agree)

- Number of steps taken per day
- Heart rate and / or other physiological data
- Sound recordings: recordings of conversations

- Sound recordings: recordings of your surroundings / ambient noises
- Information about places where you are or have been (eg, by using GPS data)
- Information about your sleep (eg, sleep duration, quality of sleep, day / night rhythm)
- Information on the use of apps, without content (eg, no use of messages that you have sent to friends)
- Information about using apps with specific content (eg, permission to analyze text and text length that you have sent to friends)
- Number of incoming and outgoing calls / messages / notifications
- Worries, problems, feelings, thoughts that concern you

28. How important is it for you to know who developed the app?
    - Very important
    - Important
    - Neither nor
    - Unimportant
    - Very unimportant

29. How much trust do you have in the following possible developers / providers of mHealth apps? (Carousel: Strongly disagree…Strongly agree)
    - Companies (eg, Google)
    - Independent research institutions (eg, universities)
    - Health insurance
    - State institutions (eg, Ministry of Health)

VII. Covid-19

30. The corona pandemic is of great significance for the current situation. With the following questions, we want to find out more about how the recent situation is for you personally. (Grid Scale: Strongly agree…strongly disagree)
    - I'm worried about the coronavirus
    - I feel personally endangered by the coronavirus
    - I fully support government measures to slow the spread
    - I feel severely restricted by government measures to slow down the virus spread.

30.1 How often do you miss the company of others?
- Never
- Very rarely
- Rarely
- Occasionally
- Often
- Very often

30.2 How often do you feel socially isolated?
- Never
- Very rarely
- Rarely
- Occasionally
- Often
- Very often

31. Since the outbreak of the corona virus pandemic, how often have you felt... (Carousel: Never…Very often)

- ...exhausted for no apparent reason?
- ... nervous?
- ... so nervous that nothing could calm you down?
- ... hopeless?
- ... restless or fidgety?
- ...so restless you couldn't sit still?
- ... down?
- ... so depressed that nothing could cheer you up?
- ... so that everything means an effort for you?
- ... worthless?

VIII. Current Worries

32. On a scale from 1 (not at all worried) to 7 (very worried), how worried have you been in the past week that... (Grid Scale: 1…7)

- ... small businesses in your city close permanently/file for bankruptcy?
- ... the gap between rich and poor is widening?
- ... the healthcare system is overburdened?
- ... society is becoming more selfish?
- ... you lose someone who is important to you?
- ... you get into financial difficulties?
- ... there will be limited access to food?
- ... you lose your job or cannot complete your (school) training/degree?
- ... you cannot meet the requirements (working from home, home schooling)?
- ... will your living situation deteriorate in the near future?

33. The novel coronavirus is something I...

- ...never think about
- ...don't think about often
- ...am neutral about
- ...tend to think frequently about
- ...keep thinking about

34. For me, the novel coronavirus is...

- ... not scary
- ...rather not scary
- ...neutral
- ...rather scary
- ...scary

35. For me, the novel coronavirus is...

- ...not worrying
- ...rather not worrying
- ...neutral

- ...rather worrying
- ...worrying

IX. Use of mhealth apps during Covid-19 pandemic

36. Are there things/activities that help you to feel better in the current situation?

- Yes
- No

What things are helping you the most right now?

____

37. Do you think that an mHealth app could help you dealing with the corona situation?

- Strongly disagree
- Disagree
- Neither nor
- Agree
- Strongly agree

38. What trainings that can be offered by an mHealth app, could help you deal better with the corona situation at the moment? (Carousel: Strongly disagree…Strongly agree)

- Relaxation, meditation and mindfulness exercises
- Support in stress management
- Prompts to be physically more active (sports)
- Measurement of physiological/physical activity (heart rate, pedometer)
- Help with daily structure/time management
- Help with nutrition / diet
- Opportunity to share health information with others (online forums)
- Support for sleep duration or sleep quality
- Write down feelings, thoughts or activities (digital diary)
- Information about health-related topics

39. Are there any offers of help/content that would help you but have not yet been mentioned?

- Yes
- No

39.1 Please tell us the offers/content that would help you:

____

X. Appreciation for Participation, Incentives, Further contact options

(eg, for questions / concerns regarding the questionnaire, further interest in research)
