# Supplementary material for: Artificial intelligence-informed mobile mental health apps for young people: a mixed-methods approach on users’ and stakeholders’ perspectives
Source: Child Adolesc Psychiatry Ment Health. 2022 Nov 17;16:86. doi: 10.1186/s13034-022-00522-6 (PMC9672578; doi:10.1186/s13034-022-00522-6)
Supplement: Supplementary file 4 — Additional file 4: Overview of participants in the qualitative study. [file 13034_2022_522_MOESM4_ESM.pdf]

Multimedia Appendix 4

Overview of participants in the qualitative study

Expert Interviews

ID	Date of interview	Field of work	Position
EXP1	18.03.2020	Psychologist	Head of school psychological counseling center
EXP2	23.03.2020	Software developer	Expert in medical technology and mobile health applications
EXP3	26.03.2020	Social pedagogue	Managing director of non-governmental organization for youth participation
EXP4	26.03.2020	Media educator / pedagogue	Head of media center
EXP5	30.04.2020	Psychologist	Head of psychological counseling center

Focus Groups

ID	Date of interview	Group	Age	Gender	Education	Job title
P1, FG1	25.03.2020	1	17	female	currently attending Gymnasium[1]	Student/Pupil, Gymnasium[1]
P2, FG1	25.03.2020	1	20	male	Abitur[2]	Part time job in retail; waiting for start of semester / university
P3, FG1	25.03.2020	1	17	female	currently visiting Gymnasium[1]	Student/Pupil, Gymnasium[1]
P4, FG1	25.03.2020	1	20	male	Abitur[2]	Part time in Software Development; waiting for start of semester / university
P1, FG2	16.04.2020	2	20	female	Abitur[2]	Student in economics
P2, FG2	16.04.2020	2	20	female	Abitur[2]	Student in medicine
P3, FG2	16.04.2020	2	15	male	currently visiting Gymnasium[1]	Student/Pupil, Gymnasium[1]
P4, FG2	16.04.2020	2	20	male	Abitur	Student in economics

[1] Secondary school leading to the “Abitur”. Prepares students for higher education at a university, comparable to preparatory high school in the US.

[2] Abitur = diploma corresponding to university entrance level, equivalent to the baccalauréat (FR, LT), the matura (PL, AT), etc.
