# Supplementary material for: Artificial intelligence-informed mobile mental health apps for young people: a mixed-methods approach on users’ and stakeholders’ perspectives
Source: Child Adolesc Psychiatry Ment Health. 2022 Nov 17;16:86. doi: 10.1186/s13034-022-00522-6 (PMC9672578; doi:10.1186/s13034-022-00522-6)
Supplement: Supplementary file 6 — Additional file 6: Coding Trees. [file 13034_2022_522_MOESM6_ESM.pdf]

Coding Trees[1,2]

Focus Groups: Coding Tree

FIRST LEVEL	SECOND LEVEL	THIRD LEVEL
K1: Concepts of "mental health"	K1_U1: Successful handling of challenges	-
	K1_U2: Awareness / understanding of physical and mental condition	-
K2: Activities to promote one's mental health	K2_U1: Meditation	-
	K2_U2: Crafting / meditative activity(s)	-
	K2_U3: Physical activity	-
	K2_U4: Being alone / withdrawal	-
	K2_U5: Social contacts	-
	K2_U6 Paying attention to nutrition	-
	K2_U7 Paying attention to sleep and waking times	-
	K2_U8 Distancing from technology / technologies	-
	K2_U9 Uncertainty / no strategies	-
K3: Use of "health-promoting apps"	K3_U1: Current usage of certain apps	K3_U1_UU1: Sports and Fitness
		K3_U1_UU2: Sleep Optimization
		K3_U1_UU3: Meditation and mindfulness
		K3_U1_UU4: Others
	K3_U2: Reasons for no longer using such apps	K3_U2_UU1: App goal met - no need anymore
		K3_U2_UU2: Unreliability of the app
		K3_U2_UU3: Too tiring to use
		K3_U2_UU4: App unsuitable for desired goal
		K3_U2_UU5: Detail / structure of the app was annoying
		K3_U2_UU6: Payment model as an obstacle to continue using the app
		K3_U2_UU7: In general, apps not of particular interest

[1] The data analysis was conducted in German. The coding trees were later translated to English.
[2] The coding trees contain all categories from data analysis. Emerged subthemes are depicted in level 2 and, if further differentiation was possible, also in sublevel 3. Not shown here are codes and codings, which exceeds the scope of this overview.

FIRST LEVEL	SECOND LEVEL	THIRD LEVEL
	K3_U3: Reasons for using an app in the past	K3_U3_UU1 Just trying out (without "goal")
		K3_U3_UU2 Problem/Goal-oriented use of the apps
K4: Use of health-related Apps in the circle of friends and B	-	-
K5: Concept of artificial intelligence	-	-
K6: Knowledge about the use of artificial intelligence in everyday life	K6_U1: Use of AI in advertising	-
	K6_U2: Use of AI in social media	-
	K6_U3: Use of AI in various contexts	-
	K6_U4: Use of AI theme in science fiction	-
K7: Attitude towards the use of AI in general	K7_U1: Critical, negative attitude, skepticism	-
	K7_U2: neutral / conditionally positive: open but critical attitude	-
	K7_U3: positive, expectant: surprised about technical possibilities	-
	K7_U4 No statement	-
K8: Questions/ambiguities regarding the input of the planned AI app	-	-
K9: Feedback on the AI4U-App after input	K9_U1: Emphasis on customizability of the app	
	K9_U2: Emphasis on the broad tableau of exercises	-
K10: Concerns about the use of existing AI	K10_U1: Concerns about the use of the AI4U app	K10_U1_UU1: No / few concerns about AI use
		K10_U1_UU2: major concerns about using the present AI APP

FIRST LEVEL	SECOND LEVEL	THIRD LEVEL
	K10_U2: Shareable information	K10_U2_UU1: Requirements for sharing information
		K10_U2_UU2: (Un)critical information
		K10_U2_UU3: Doubts about functionality for major mental health issues
K11: Wishes / ideas / suggestions for the app	K11_U1: Possible difficulties / problems of an mHealth app	K11_U2_UU1: Too much effort for use
		K11_U2_UU2: App makes inaccurate/bad suggestions
		K11_U2_UU3: Important: control over personalization options.
	K11_U2: Desired exercises / trainings / functions	K11_U2_UU1: App provides an overview of possible exercises
		K11_U2_UU2: App suggests solutions in difficult situations
		K11_U2_UU3: App provides background information for exercises
		K11_U2_UU4: No sharing of exercises / achievements with others
		K11_U2_UU5: No sharing of problems/issues with others
		K11_U2_UU6: App suggests distance to mobile phone
K12: (Types of) collaboration on the app	K12_U1: Interested in working	-
	K12_U2: Possibilities / type / idea of participation	-

Expert Interviews: Coding Tree

FIRST LEVEL	SECOND LEVEL	THIRD LEVEL
K1: Professional position / Field of work	-	-
K2: Mental health promotion (MHP) and mental disorder prevention in young people (YP) in the professional context of the expert	K2_U1: Terminology / Definition of Mental Health Promotion	-
	K2_U2: Current status / relevance of MHP in YP	-
K3: Relevance of apps for MHP in YP from the expert's point of view	K3_U1: Current importance and nature of apps	-
	K3_U2: Problems / barriers for apps	-
K4: Input for creating an app with AI for MHP in YP	K4_U0 Take into account the need structure of YP	-
	K4_U1 Forwarding YP to help centers for "bigger problems" / risks	-
	K4_U2 Target group-oriented approach / language	-
	K4_U3 Character / form of possible interventions in the app	K4_U3_UU1 Give stories / everyday examples
		K4_U3_UU2 App gives recommendations and ideas
		K4_U3_UU3 Initiation of self-reflection
		K4_U3_UU4 Interventions to experience self-efficacy
	K4_U4 Content of possible interventions in the app	K4_U4_UU1 Psychoeducation / knowledge transfer
		K4_U4_UU2 Emotion regulation
		K4_U4_UU3 Self-acceptance / self-worth
		K4_U4_UU4 Physical activity
		K4_U4_UU5 Improve social skills / dealing with others
K5: Chances of apps with AI for MHP in YP	K5_U1 App as low-threshold access to YP	-
	K5_U2 App supports search for help and treatment	-
	K5_U3 Reaching risk groups early on	-

FIRST LEVEL	SECOND LEVEL	THIRD LEVEL
	K5_U4 App as a holistic solution	-
K6: Limits of / risks in apps with AI for MHP in YP	K6_U1 No risks / few concerns	-
	K6_U2 (Possible) Risks for YP	K6_U2_UU1 Risks to enforce self-optimization in YP
		K6_U2_UU2 Risks from difficult topics
		K6_U2_UU3 Risks from financial interests
K7: Participation of practitioners in the creation of the app	K7_U1 Requirements for participation of practitioners	-
	K7_U2 Focus for individual participation	-
	K7_U3 Suggestions for the inclusion of other practitioners	-
	K7_U4 Input in preliminary/ application phase	-
K8: Prospective development of MHP of YP (in 5 years)	K8_U1 Integration of AI and MHP into school lessons	-
	K8_U2 AI-Apps as a supplement to real consulting offers	-
	K8_U3 Other implementation options of the app	-
